# Supplementary material for: AI-powered simulation-based inference of a genuinely spatial-stochastic gene regulation model of early mouse embryogenesis
Source: PLoS Comput Biol. 2024 Nov 14;20(11):e1012473. doi: 10.1371/journal.pcbi.1012473 (PMC11614244; doi:10.1371/journal.pcbi.1012473)
Supplement: S1 Appendix — Summary of inferred model parameter interactions (posterior distribution). General considerations for model parameter inference. Reflection and outlook. (PDF) [file pcbi.1012473.s001.pdf]

AI-powered simulation-based inference of a genuinely spatial-stochastic gene regulation model of early mouse embryogenesis [S1 APPENDIX]

Michael Alexander Ramirez Sierra[1,2,*], Thomas R. Sokolowski[1]

1 Frankfurt Institute for Advanced Studies (FIAS), Frankfurt am Main, Germany
2 Faculty of Computer Science and Mathematics, Goethe-Universität Frankfurt am Main, Frankfurt am Main, Germany

* ramirez-sierra@fias.uni-frankfurt.de

Supporting information

Summary of inferred model parameter interactions (posterior distribution)

We summarize the model parameter values estimated using our SNPE-powered workflow. We present the full inferred model parameter distribution, as well as the first estimate of the model parameter sensitivity. In S1 and S2 Figs, the unconditional (odd rows) and the conditional (even rows) posterior parameter distributions are split into multiple distinct blocks grouping parameters according to their particular regulatory roles in the ICM development process. This split is helpful for showing the critical model components and the correlations among the most concomitant inferred parameters. For each block, the diagonal entries are the marginal (one-dimensional) distributions, and the upper-diagonal entries are the joint (two-dimensional) distributions between successive parameters. For obvious reasons, other higher-dimensional projections are not displayed. See Table 4 for a recap of the inferred model parameter values and their corresponding notation.

Concentrating on the original inferred system (ITWT), we now examine the posterior distribution of its model parameters. The first pair of parameter blocks (Fig S1A and S1C) displays two closely associated distributions for the core GRN motif components: the unconditional posterior (top row) shows the comparatively short span over the parameter space which enables the system to display the postulated target behavior; the conditional posterior (bottom row) shows at marginal and joint levels the sensitivity to variations of these parameter values. To correctly analyze this last posterior, we must initially recognize that this distribution is indeed the raw inferred posterior, but it is now conditioned on the maximum-a-posteriori (MAP) estimate of all the model parameters. In that sense, every diagonal entry or marginal distribution reflects the tolerance to fluctuations of a single value, given that all the other parameter values are fixed at its corresponding MAP estimate. In other words, we automatically get an approximation of the sensitivity for every inferred model parameter conditional on the MAP estimate of the remaining values; this feature is inherent to the SBI technique used here, without the need for additional expensive simulations. Likewise, we can perform an analogous assessment for every upper-diagonal entry or joint distribution based on the same criteria. In this case, we see that the core GRN motif values are constrained to a small parameter space region, which emphasizes their sensitive balancing act and their importance for attaining the ideal score.

Similarly, the second pair of parameter blocks (Fig S2A and S2E) shows the distributions linked to the interactions among NANOG, GATA6, FGF4, and ERK. Especially for the *Ffg4* gene, the span of possible parameter values pertaining to its regulation is relatively broad.

Nevertheless, when the other parameters are fixed appropriately, the sensitivities of *Fgf4*_NANOG (half-saturation threshold for transcriptional activation of *Fgf4* promoter by NANOG) and *Fgf4*_GATA6 (half-saturation threshold for transcriptional repression of *Fgf4* promoter by GATA6) increase considerably, stressing their significance for the target system-model behavior. In contrast, for the case of *Gata6*_A-ERK (half-saturation threshold for transcriptional activation of *Gata6* promoter by A-ERK) and *Nanog*_A-ERK (half-saturation threshold for transcriptional repression of *Nanog* promoter by A-ERK), we see relatively low sensitivities; hence, these parameters could potentially be adjusted easily without affecting the ideal score and the final cell-fate ratio.

In the same manner, the third pair of parameter blocks (Fig S2B and S2F) shows that the values for signaling components can be drawn from a rich and large parameter space region without affecting the target behavior of the underlying system. This property clearly only holds provided that cell-cell communication is fully operational, because its absence will cause the system model to materialize almost exclusively high-NANOG- and low-GATA6-expressing cells.

Comparably, the fourth pair of parameter blocks (Fig S2C and S2G) displays a set of rather broad spans for the phosphorylation and dephosphorylation parameters relevant to the FGF-ERK pathway that propagates the external FGF4 signal back into the cell, except for $\tau_{\mathrm{doh,ERK}}$, the half-turnover time for the inactivation of ERK. The fast inactivation rate that we find here might indicate that the system relies on quick adaptations to the amount of extracellular signaling.

Lastly, the fifth pair of parameter blocks (Fig S2D and S2H) shows several interesting relationships. The two parameters "Mean Initial mRNA Count" and "Mean Initial PROTEIN Count" are noticeably anti-correlated in a linear fashion. This fact plausibly indicates that the neural network learnt the complementary interaction between these two parameters, which have a conjoint effect on the initial expression dynamics of the three main genes (*Nanog*, *Gata6*, and *Fgf4*). This complementary interaction itself reflects the need for constraining these two values to a region where they will not exceed the theoretical maximum mean copy numbers per cell of mRNA and protein molecules for the key players at the start of the simulations, in our case 250 mRNA (*Nanog*-*Gata6*) and 1000 protein (NANOG-GATA6) copies (see subsection "Model at cell scale" of "Materials and Methods") . For information about the definite usage of these two parameters to create the initial condition distributions for model simulations, see "Computational experiments". In the same way, we see that the restricted value range of the parameter $\tau_{\mathrm{d,M\text{-}FGFR\text{-}FGF4}}$ might indicate that, once a substantial amount of FGF4 escapes the cytoplasm and binds to the available FGFR population on the cellular membrane, the FGFR-FGF4 complex-monomer lifetime must be tightly controlled as to not over-amplify the signal or to compromise the buffering of FGF4 fluctuations.

General considerations for model parameter inference

Biophysically-realistic mechanistic modeling is integral to achieving a quantitative understanding of the behavior of complex biological systems. These biophysical representations are fundamentally generative models: they aim at providing a mechanistic description of the underlying biological phenomenon, enabling the generation of temporally-faithful synthetic trajectories of the given modeled dynamics, which potentially resemble empirical findings or target observations. As such, these models require a rich collection of experimental data detailing the principal mechanisms and physical processes triggering the phenomenon under study [1].

For early developmental biology systems, in particular, experimental studies typically can not simultaneously measure all pivotal biophysical variables, and they generally can not capture enough granular features to facilitate comprehensive mechanistic modeling; especially, considering the complex GRNs and signaling pathways coordinating the proper progression of these systems, as well as their inherent nonlinear-multiscale dynamics. While these models are challenging to construct, we here demonstrate that despite the lack of detailed quantitative measurements, empirical qualitative observations can provide suitable bases for biophysical mechanistic modeling.

In that respect, formulating such a nonlinear-multiscale representation of the underlying biological system is only the preparatory step for model building. Arguably, the greatest challenge of biophysical mechanistic modeling is the estimation of suitable parameter values which allow the inferred model to recapitulate the most fundamental characteristics of the studied system behavior.

A popular method known as approximate Bayesian computation (ABC) has been widely used in computational biology for inferring sensible model parameter sets within the context of biophysical mechanistic modeling [2]. ABC is itself a collection of modern algorithms which essentially compare experimental (target) and synthetic (simulation) data based on a given metric or distance function, measuring discrepancies between these two data vectors via the involvement of predetermined summary statistics. What is more, the family of ABC algorithms empowers the applicability of statistical inference to situations where the likelihood function of a stated model is intractable, enabling an approximation of the true posterior distribution of the respective parameter values.

However, an AI-powered SBI framework has recently emerged as an influential technique to tackle any likelihood-free inferential challenge [2]. In general, SBI does not attempt to retrieve the single best parameter set. Instead, SBI helps to discover high-probability parameter space regions capable of explaining the target observations, while potentially facilitating the quantification of parameter uncertainty [3].

Even though these AI-centered techniques are similar to the ABC methods, they provide significant benefits over their prevalent counterparts: they are innately flexible, which allow them to tackle a broad range of problems; they are structurally designed to learn low-dimensional representations of high-dimensional datasets, but trading off interpretability against predictive power of the stated model; they can in principle dramatically improve inference efficiency because they demand relatively small training sets, generating additional synthetic data from the learned latent space of the ANN; they automatically offer an estimate of the model parameter uncertainty, which does not need supplemental simulations. Particularly, the sequential neural posterior estimation (SNPE) procedure completely skips the creation of a virtual likelihood function, and it explicitly approximates the model parameter posterior distribution [4, 5].

In this work, to overcome our challenging inferential task and inspired by the simulation-based inference (SBI) framework, we exploit this novel AI-powered parameter estimation approach and combine it with various classical ML techniques. At its core, our inference approach directly exploits simulation data for exploring parameter space, yet it indirectly relies on primarily qualitative data to inform/constrain the parameter values; the simulation data is accordingly generated via the proposed spatial-stochastic model of mouse ICM lineage differentiation. Our model parameter estimation scheme is thus guided by the SBI paradigm [2], and its centerpiece is the sequential neural posterior estimation (SNPE) algorithm [4, 5].

While this novel family of Bayesian inference algorithms has a huge application potential, these schemes are typically difficult to implement, requiring access to big computational resources. These contemporary methods have been mostly adopted in neuroscience research [2, 6–8], so far having a limited reach within the broad field of computational biology (and developmental biophysics in particular).

Preliminary model design

The preparatory stage is establishing the elementary structure of the model. Our primary goal is to integrate two basic building blocks: a cell-scale submodel which consists of the GRN coordinating the mouse ICM lineage specification process; a tissue-scale submodel which describes the cell-cell signaling interactions. Another pivotal goal is to create a minimal spatial-stochastic description of the developing mouse ICM. This minimal model should enable exact simulation of the proposed system dynamics. Therefore, only the most relevant biochemical species prevailing in the underlying biological system should be part of this minimal model (see Table 1). On the flip side, other purely-computational species might be necessary for correctly analyzing and tracing the intricate simulated dynamics.

An additional basic assumption concerns the ICM spatial configuration. In our case, the full neighborhood representation is a rectangular voxel grid with a one-cell thickness, where each voxel emulates an embryo cell. This static cell arrangement isolates our analysis from other dimensions of variability, and it focuses the study to the question of how a cell-cell signaling mechanism might create the so-called “salt-and-pepper” pattern [9–12].

Altogether, biophysical mechanistic modeling is expected to give us a panoramic insight into the central mechanisms causing the phenomena under study. Ideally, this enriched understanding will generate testable predictions, guiding innovative experiments which pursue related processes. Although, systematically developing these models is a complex and nontrivial problem in computational biology [13].

Basic theoretical assumptions

To quantitatively model processes such as gene expression dynamics and cell signaling pathways, the Chemical Master Equation (CME) is a popular approach. Furthermore, to incorporate reaction-diffusion processes, these biochemical networks are preferably abstracted using the Reaction-Diffusion Master Equation (RDME) formalism. In that regard, these two frameworks represent biochemical phenomena as a network system involving multiple reactions (edges) and species (vertices or nodes), where each edge has an associated rate related to the propensity function of the given reaction [14]. Along with it, while balancing the relationship between computational efficiency and biophysical realism, the RDME fuses the notion of spatial heterogeneity or partitioning with the ingrained stochasticity of the CME.

Such stochasticity or noise plays a focal role for multifold cellular processes [15, 16]. One particularly important example is the cell-fate decision-making process: even when genetically identical cells are subject to homogeneous (equivalent) experimental conditions, they manifest significant gene-expression variability [17,18]. In fact, this cell-differentiation process is deemed to be a truthful reflection of the underlying gene regulatory network (GRN) dynamics. In a general sense, a central GRN not only must be capable of perpetuating a stable/attractor functional state, but it also must be sensitive to external stimuli in order to adapt the genetic program of a cell according to its environment, allowing it to reach other stable/attractor functional states [19]. These factors ultimately permit a cellular population to coordinately achieve some globally-conserved pattern of lineages.

Within this context, two contrasting perspectives exist to interpret cellular patterning heterogeneity. The classical perspective views gene expression and cell-fate decision making as fundamentally deterministic mechanisms where noise has a mainly passive role; in this case, the phenomenon of random molecular/cellular count fluctuations is assumed to be an additive (or

sometimes multiplicative) noise process. Instead, we adopt in this work the other "contemporary" perspective: cell-cell variability encapsulates highly-valuable information about the genetic programs orchestrating cellular-function specification, which itself manifests the inherently noisy nature of the underlying gene expression regulation [19]. This stochasticity-controlled perspective (where noise has a structurally active role) is thus attained via the RDME formalism, which in turn facilitates the inclusion of spatial/compartmental modeling elements.

Basic empirical observations

A central idea of biochemical network model building is to reproduce some particularly interesting behaviors, phenotypes, or empirical observations [20]. Nevertheless, because of highly-nonlinear interactions among genetic regulatory components, a single set of experimental data by itself can not uncover the full functional dynamics of such biological systems [17]. It is hence normally necessary to consolidate several datasets coming from multiple related systems under diverse experimental conditions; for this reason, coherent and viable data analysis becomes a considerably difficult endeavor on its own. What is more, these quantitative observations often do not provide enough details for spatial-stochastic modeling, and they are typically incomplete: from the practical point of view, it is extremely challenging to provide simultaneous measurements for all the key elements dictating the underlying multiscale dynamics.

However, we can exploit any (high-level) mostly-qualitative experimental observation for successfully constraining/informing our model parameter inference approach. For the developing mouse blastocyst and its related experimental systems, there are three determining characteristics which describe the ICM fate-specification process: (1) the two cell lineages emerging from the ICM exhibit highly-reproducible proportions (ratio of 2 : 3 for EPI and PRE fates) [21, 22]; (2) the formation of the blastocyst takes approximately 1.5-2 days of embryonic development [23–25], and (more crucially) EPI-PRE populations should reach their expected fate proportions by the 40-hour time mark (roughly 8 or 12 hours before the end of the preimplantation period) [25, 26]; (3) the absence of FGF4 signaling forces the ICM to almost exclusively adopt an EPI fate (the ICM population is naturally biased towards a naive cellular pluripotency) [27, 28].

The last characteristic is notably important because it underlines the key role that cell-cell signaling plays for correct ICM patterning formation. This feature also highlights a critical difference between the two inferred system models: for replicating the target behavior of the underlying biological system (final EPI-PRE proportions), the ITWT model should involve a cell-nonautonomous mechanism and the RTM model should depend on a cell-autonomous process. In other words, within the whole ICM population, the probability of a given cell adopting a specific lineage should be conditional on the fates taken by other cells for the ITWT, and it should be independent of the fates taken by other cells for the RTM.

Reflection and outlook

The specification of the inner cell mass (ICM) lineages is a pivotal process in mouse blastocyst formation and an important paradigm in tissue development. During this process, two distinct cell lines, the epiblast (EPI) and the primitive endoderm (PRE), differentiate in a reliable manner without any dependency on maternal inputs, unfolding from the zygote in a completely self-organizing fashion. To this end, the embryo orchestrates multiple subprocesses at two ancillary spatio-temporal scales: at the single-cell level, complex regulatory interactions concertedly calibrate genetic programs, partly responding to membrane-receptor mediated feedback loops that can couple them to neighboring ICM cells; at the tissue level, globally conserved features materialize driven by biochemical signaling throughout the system.

The stochastic character of gene expression dynamics, together with the relatively small number of cells forming the early mouse embryo, make ICM differentiation an inherently noisy process. Therefore, correct progression of ICM specification not only depends on a stochastic surge of the EPI and PRE fates, but also leans on reliable cellular maturation of these arising lineages. Maintaining a well-balanced ratio between EPI and PRE populations is particularly important in this context, as breaking this balance can have significant physiological implications for the early mouse embryo [22,24]. A successful conclusion of these processes requires mechanisms that make the developing tissue robust against intrinsic noise and extrinsic perturbations. Recent studies have shown that an FGF-mediated cell-cell communication mechanism constitutes a necessary precondition for the robust emergence of the two distinct ICM lineages, seemingly adding a deterministic dimension to this process [29, 30].

The inherent presence of randomness in ICM differentiation is evidenced by the significant cell-cell heterogeneity observed in experiments [25, 31–33], and reproducing these characteristics sets a benchmark for any faithful model of this system. Therefore, a genuinely stochastic modeling approach that realistically incorporates the noisy dynamics of gene regulatory networks (GRNs) and cellular signaling pathways is necessary for understanding cell fate specification during early mouse development, as well as for quantifying its robustness.

Several phenomenological models have been proposed for blastocyst formation in the mouse embryo [21, 29, 34, 35], but they are primarily deterministic in nature, and as such do not allow for a rigorous investigation of the implications of noise emerging from the basic processes driving this developmental process, neither for quantification of its robustness. To correctly capture the inherent randomness in ICM differentiation, we developed a biophysics-rooted spatial-stochastic model simulated via the Reaction-Diffusion Master Equation (RDME) formalism, and embedded it into a Simulation-Based Inference (SBI) framework building on recent advancements in Machine Learning (ML). Our multi-cellular model mechanistically describes the biochemical ICM patterning dynamics and its accuracy using biologically realistic lifetimes for the involved molecular species, and provides a biophysically realistic implementation of the mesoscopic processes generating noise at the cell level. Using this combined framework, we inferred multiple parameter distributions that inform our model both in wild-type-like and several mutant-like conditions.

In summary, our genuinely spatial-stochastic modeling approach, which realistically incorporates the noisy dynamics of gene regulatory networks (GRNs) and cellular signaling pathways, opens the necessary venue for understanding cell-fate specification and proportioning principles during early mouse development as well as similar biophysical systems. This is because our approach enables the quantification of robustness and reproducibility for target system behaviors in the presence of biophysically grounded intrinsic noise alongside extrinsic perturbation sources.

References

1. Torregrosa G, Garcia-Ojalvo J. Mechanistic models of cell-fate transitions from single-cell data. Current Opinion in Systems Biology. 2021;26:79–86. doi:10.1016/j.coisb.2021.04.004.
2. Cranmer K, Brehmer J, Louppe G. The frontier of simulation-based inference. Proceedings of the National Academy of Sciences. 2020;117(48):30055–30062. doi:10.1073/pnas.1912789117.
3. Tejero-Cantero A, Boelts J, Deistler M, Lueckmann JM, Durkan C, Gonçalves PJ, et al. sbi: A toolkit for simulation-based inference. Journal of Open Source Software. 2020;5(52):2505. doi:10.21105/joss.02505.
4. Greenberg DS, Nonnenmacher M, Macke JH. Automatic Posterior Transformation for Likelihood-Free Inference; 2019. Available from: `http://arxiv.org/abs/1905.07488`.
5. Deistler M, Goncalves PJ, Macke JH. Truncated proposals for scalable and hassle-free simulation-based inference; 2022. Available from: `http://arxiv.org/abs/2210.04815`.
6. Deistler M, Macke JH, Gonçalves PJ. Energy-efficient network activity from disparate circuit parameters. Proceedings of the National Academy of Sciences. 2022;119(44):e2207632119. doi:10.1073/pnas.2207632119.
7. Kaiser J, Stock R, Müller E, Schemmel J, Schmitt S. Simulation-based Inference for Model Parameterization on Analog Neuromorphic Hardware; 2023. Available from: `http://arxiv.org/abs/2303.16056`.
8. Tolley N, Rodrigues PLC, Gramfort A, Jones SR. Methods and considerations for estimating parameters in biophysically detailed neural models with simulation based inference. PLOS Computational Biology. 2024;20(2):e1011108. doi:10.1371/journal.pcbi.1011108.
9. Fischer SC, Corujo-Simon E, Lilao-Garzon J, Stelzer EHK, Muñoz-Descalzo S. The transition from local to global patterns governs the differentiation of mouse blastocysts. PLoS ONE. 2020;15(5). doi:10.1371/journal.pone.0233030.
10. Liebisch T, Drusko A, Mathew B, Stelzer EHK, Fischer SC, Matthäus F. Cell fate clusters in ICM organoids arise from cell fate heredity and division: a modelling approach. Scientific Reports. 2020;10(1):22405. doi:10.1038/s41598-020-80141-3.
11. Forsyth JE, Al-Anbaki AH, Fuente Rdl, Modare N, Perez-Cortes D, Rivera I, et al. IVEN: A quantitative tool to describe 3D cell position and neighbourhood reveals architectural changes in FGF4-treated preimplantation embryos. PLOS Biology. 2021;19(7):e3001345. doi:10.1371/journal.pbio.3001345.
12. Fischer SC, Schardt S, Lilao-Garzón J, Muñoz-Descalzo S. The salt-and-pepper pattern in mouse blastocysts is compatible with signaling beyond the nearest neighbors. iScience. 2023;26(11). doi:10.1016/j.isci.2023.108106.
13. Massonis G, Villaverde AF, Banga JR. Distilling identifiable and interpretable dynamic models from biological data; 2023. Available from: `https://www.biorxiv.org/content/10.1101/2023.03.13.532340v2`.
14. Pessoa P, Schweiger M, Sgouralis I, Pressé S. Accelerating likelihood calculations for biochemical network discovery. Biophysical Journal. 2023;122(3):539a. doi:10.1016/j.bpj.2022.11.2856.
15. Eldar A, Elowitz MB. Functional roles for noise in genetic circuits. Nature. 2010;467(7312):167–173. doi:10.1038/nature09326.

16. Munsky B, Neuert G, van Oudenaarden A. Using Gene Expression Noise to Understand Gene Regulation. Science. 2012;336(6078):183–187. doi:10.1126/science.1216379.

17. Schnoerr D, Sanguinetti G, Grima R. Approximation and inference methods for stochastic biochemical kinetics—a tutorial review. Journal of Physics A: Mathematical and Theoretical. 2017;50(9):093001. doi:10.1088/1751-8121/aa54d9.

18. Pang Y, Liang J. Probability landscape of a stochastic model of gene expression in single cells through exact solution of chemical master equation. Biophysical Journal. 2023;122(3):539a. doi:10.1016/j.bpj.2022.11.2857.

19. Bonnaffoux A, Herbach U, Richard A, Guillemin A, Gonin-Giraud S, Gros PA, et al. WASABI: a dynamic iterative framework for gene regulatory network inference. BMC Bioinformatics. 2019;20(1):220. doi:10.1186/s12859-019-2798-1.

20. Stillman NR, Mayor R. Generative models of morphogenesis in developmental biology. Seminars in Cell & Developmental Biology. 2023;147:83–90. doi:10.1016/j.semcdb.2023.02.001.

21. Bessonnard S, De Mot L, Gonze D, Barriol M, Dennis C, Goldbeter A, et al. Gata6, Nanog and Erk signaling control cell fate in the inner cell mass through a tristable regulatory network. Development. 2014;141(19):3637–3648. doi:10.1242/dev.109678.

22. Saiz N, Williams KM, Seshan VE, Hadjantonakis AK. Asynchronous fate decisions by single cells collectively ensure consistent lineage composition in the mouse blastocyst. Nature Communications. 2016;7. doi:10.1038/ncomms13463.

23. Płusa B, Piliszek A. Common principles of early mammalian embryo self-organisation. Development. 2020;147(dev183079). doi:10.1242/dev.183079.

24. Saiz N, Hadjantonakis AK. Coordination between patterning and morphogenesis ensures robustness during mouse development. Philosophical Transactions of the Royal Society B. 2020;doi:10.1098/rstb.2019.0562.

25. Allègre N, Chauveau S, Dennis C, Renaud Y, Meistermann D, Estrella LV, et al. NANOG initiates epiblast fate through the coordination of pluripotency genes expression. Nature Communications. 2022;13(1):3550. doi:10.1038/s41467-022-30858-8.

26. Yanagida A, Corujo-Simon E, Revell CK, Sahu P, Stirparo GG, Aspalter IM, et al. Cell surface fluctuations regulate early embryonic lineage sorting. Cell. 2022;185(5):777–793.e20. doi:10.1016/j.cell.2022.01.022.

27. Bessonnard S, Coqueran S, Vandormael-Pournin S, Dufour A, Artus J, Cohen-Tannoudji M. ICM conversion to epiblast by FGF/ERK inhibition is limited in time and requires transcription and protein degradation. Scientific Reports. 2017;7(1):12285. doi:10.1038/s41598-017-12120-0.

28. Thompson JJ, Lee DJ, Mitra A, Frail S, Dale RK, Rocha PP. Extensive co-binding and rapid redistribution of NANOG and GATA6 during emergence of divergent lineages. Nature Communications. 2022;13(1):4257. doi:10.1038/s41467-022-31938-5.

29. Saiz N, Mora-Bitria L, Rahman S, George H, Herder JP, Garcia-Ojalvo J, et al. Growth-factor-mediated coupling between lineage size and cell fate choice underlies robustness of mammalian development. eLife. 2020;9. doi:10.7554/eLife.56079.

30. Raina D, Bahadori A, Stanoev A, Protzek M, Koseska A, Schröter C. Cell-cell communication through FGF4 generates and maintains robust proportions of differentiated cell types in embryonic stem cells. Development. 2021;148(21):dev199926. doi:10.1242/dev.199926.

31. Simon CS, Hadjantonakis AK, Schröter C. Making lineage decisions with biological noise: Lessons from the early mouse embryo. WIREs Developmental Biology. 2018;7(4):e319. doi:https://doi.org/10.1002/wdev.319.

32. Ochiai H, Hayashi T, Umeda M, Yoshimura M, Harada A, Shimizu Y, et al. Genome-wide kinetic properties of transcriptional bursting in mouse embryonic stem cells. Science Advances. 2020;6(25):eaaz6699. doi:10.1126/sciadv.aaz6699.

33. Robert C, Prista von Bonhorst F, De Decker Y, Dupont G, Gonze D. Initial source of heterogeneity in a model for cell fate decision in the early mammalian embryo. Interface Focus. 2022;12(4):20220010. doi:10.1098/rsfs.2022.0010.

34. Tosenberger A, Gonze D, Bessonnard S, Cohen-Tannoudji M, Chazaud C, Dupont G. A multiscale model of early cell lineage specification including cell division. npj Systems Biology and Applications. 2017;3(1):1–11. doi:10.1038/s41540-017-0017-0.

35. Stanoev A, Schröter C, Koseska A. Robustness and timing of cellular differentiation through population-based symmetry breaking. Development. 2021;148(3):dev197608. doi:10.1242/dev.197608.
