# Supplementary figures and images for: AI-powered simulation-based inference of a genuinely spatial-stochastic gene regulation model of early mouse embryogenesis
Source: PLoS Comput Biol. 2024 Nov 14;20(11):e1012473. doi: 10.1371/journal.pcbi.1012473 (PMC11614244; doi:10.1371/journal.pcbi.1012473)

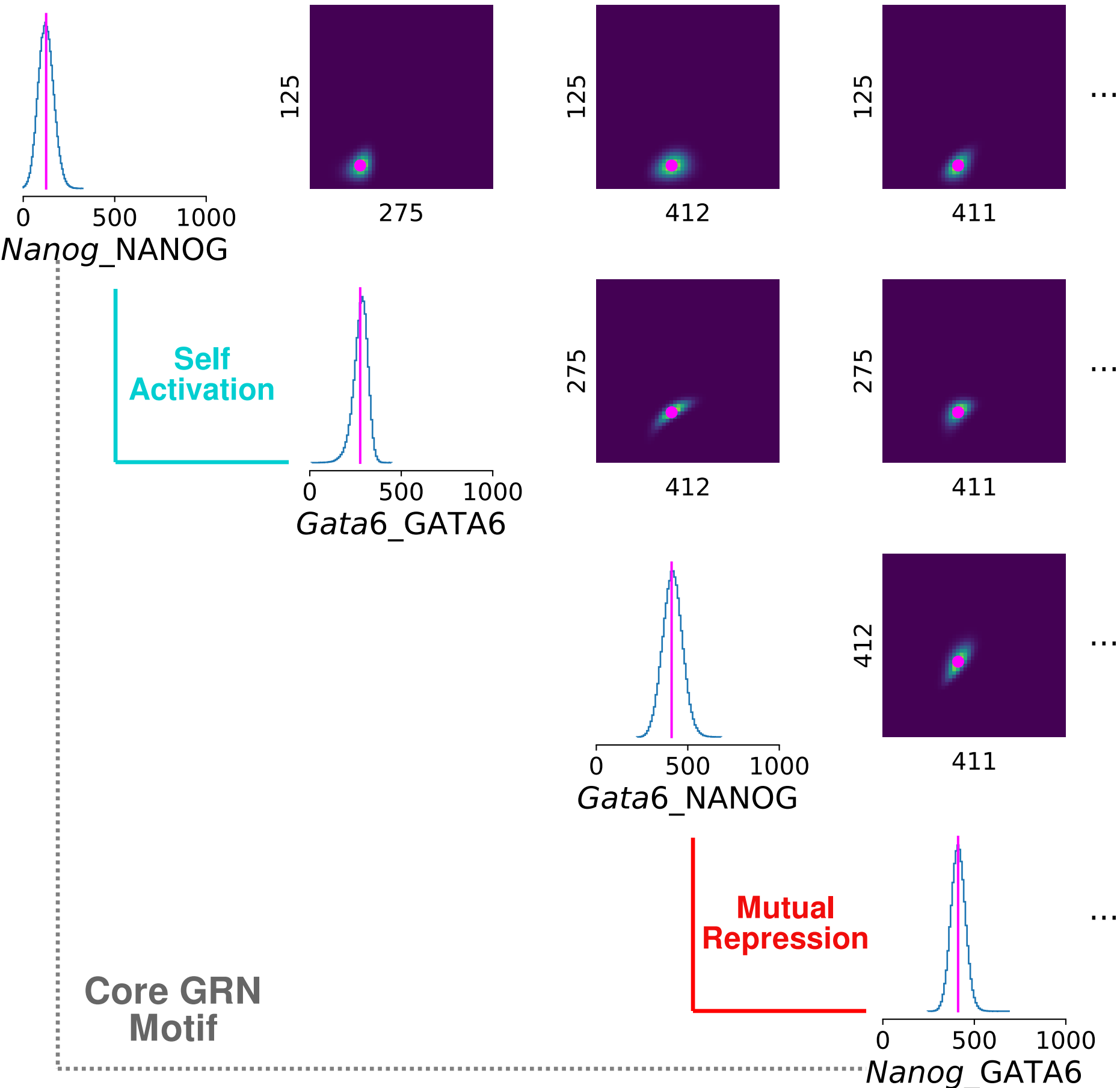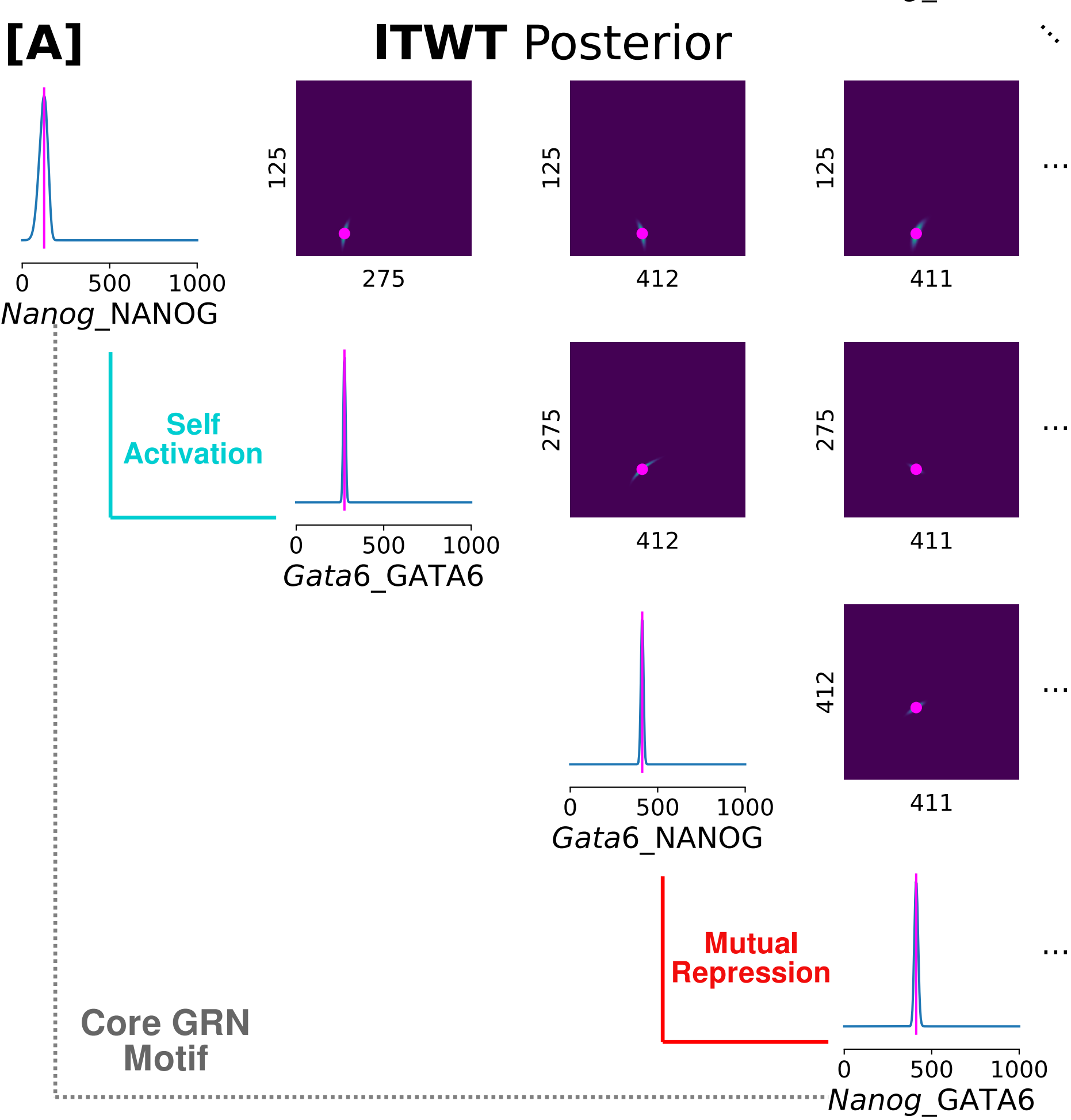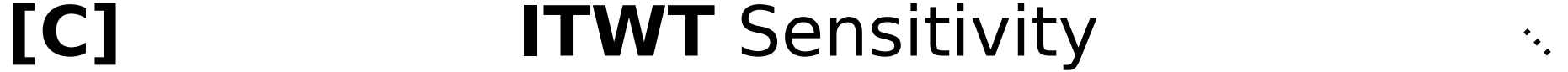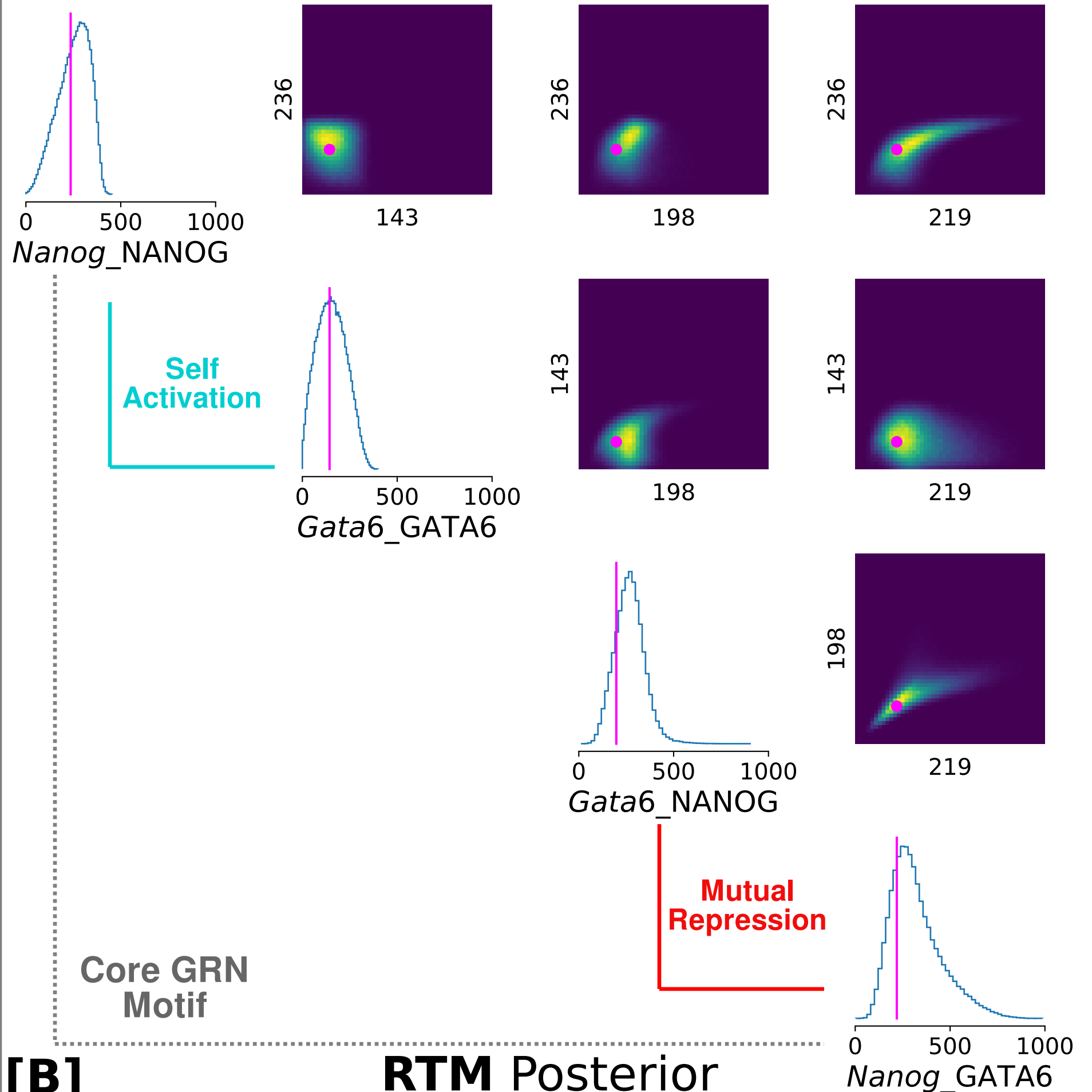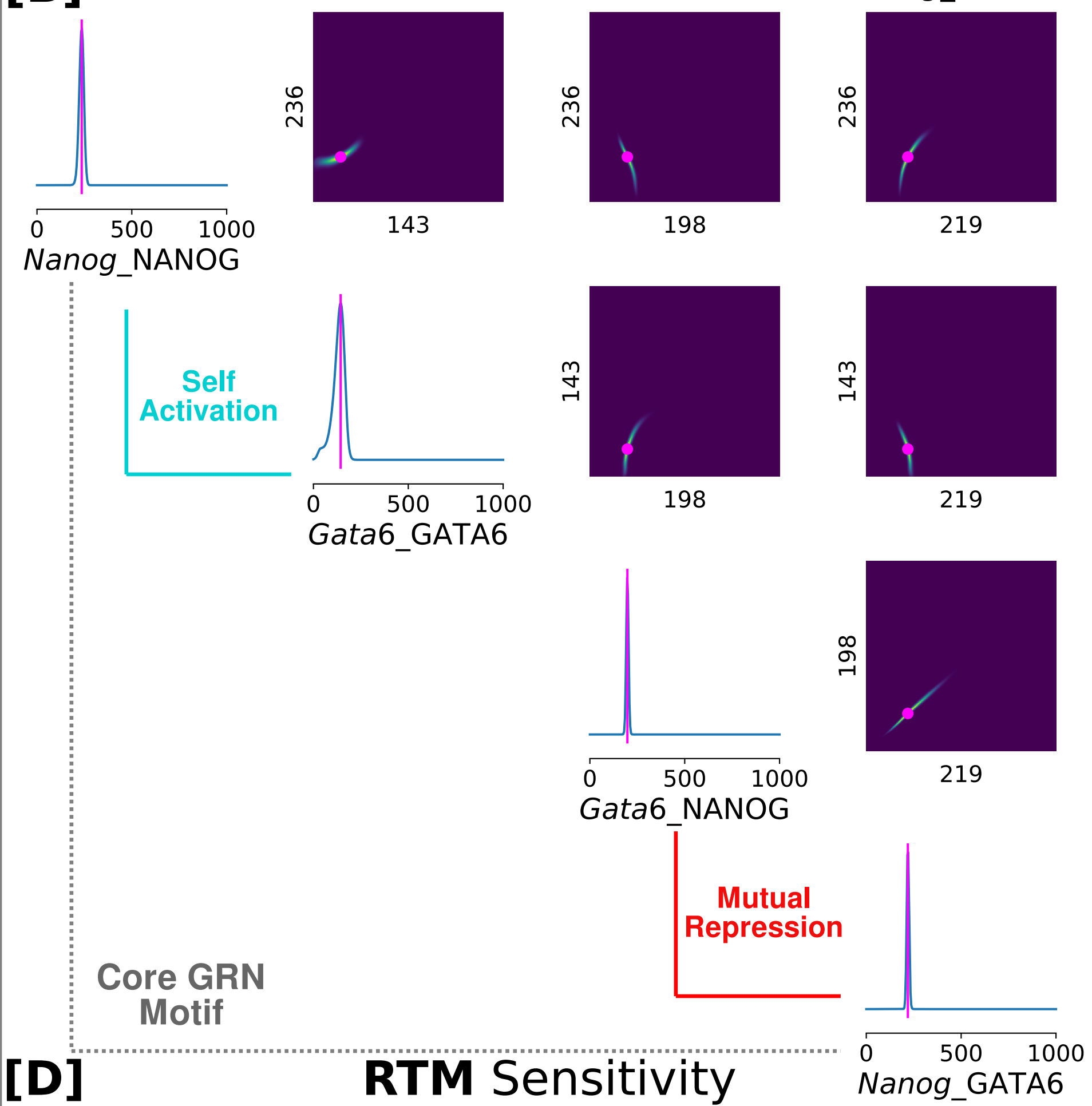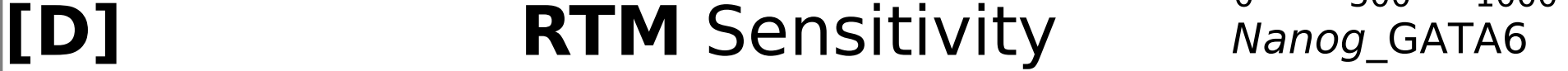

Supplement: S1 Fig — The central component of our inference scheme is the sequential neural posterior estimation (SNPE) algorithm. Both unconditional (top row [A, B]) and conditional (bottom row [C, D]) posterior parameter distributions were obtained following 8 consecutive rounds of inference. 800 thousand composite simulations were performed for the inferred-theoretical wild-type (ITWT) system (left column [A, C]). For the reinferred-theoretical mutant (RTM) system (right column [B, D]), 4 consecutive rounds of inference were performed producing 400 thousand simulations. For complete details of the model parameter inference procedure, see Model parameter inference framework. [A, B] Model parameter posterior distribution. For ease of visualization, we only show the one-dimensional projection of all posterior components representing the core GRN motif interactions. [C, D] First assessment of model parameter sensitivity. These panels show the same components as in [A, B] but the posterior is now conditioned on the maximum-a-posteriori probability (MAP) estimate of the model parameters. (PDF) [file pcbi.1012473.s002.pdf]

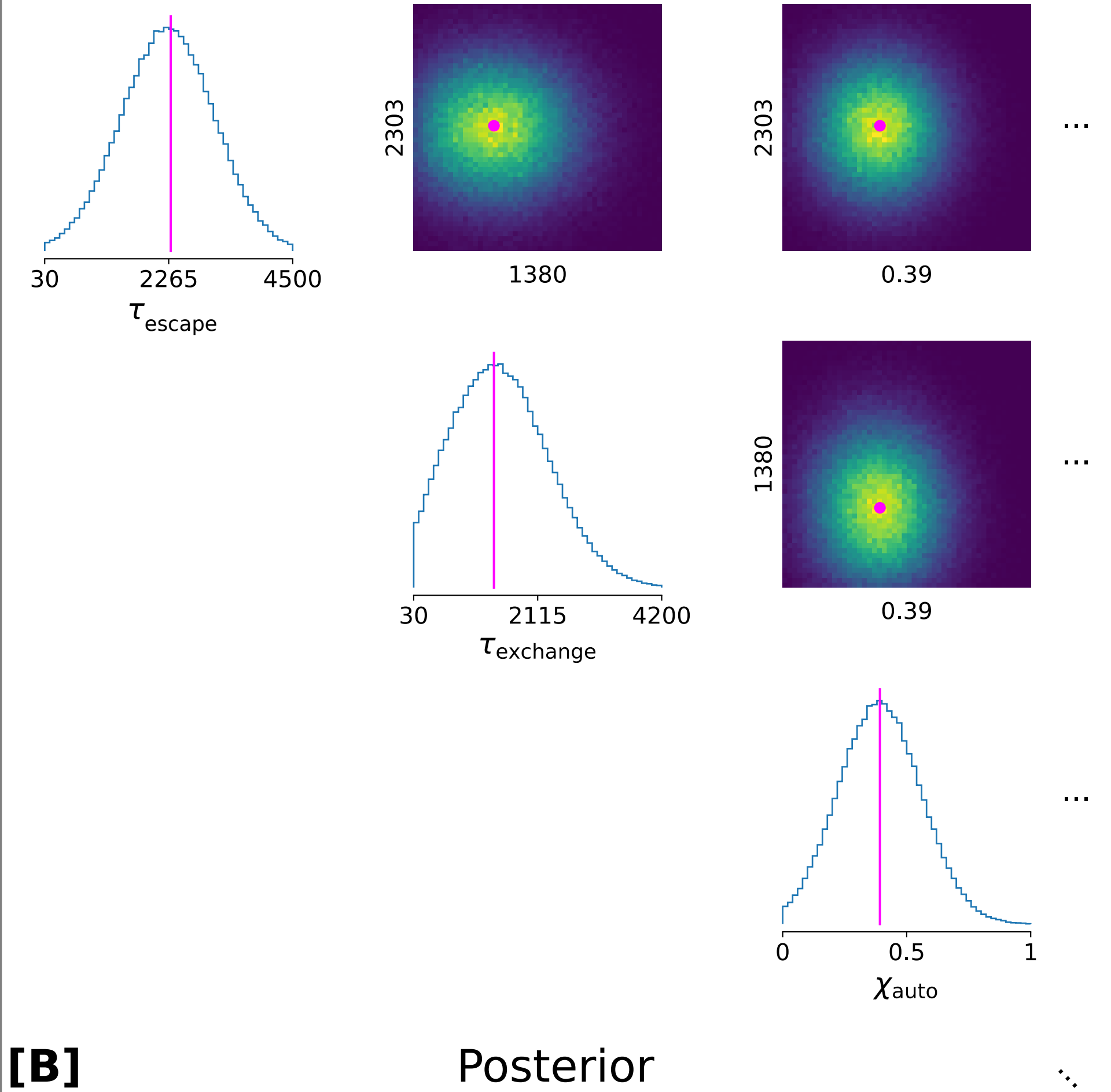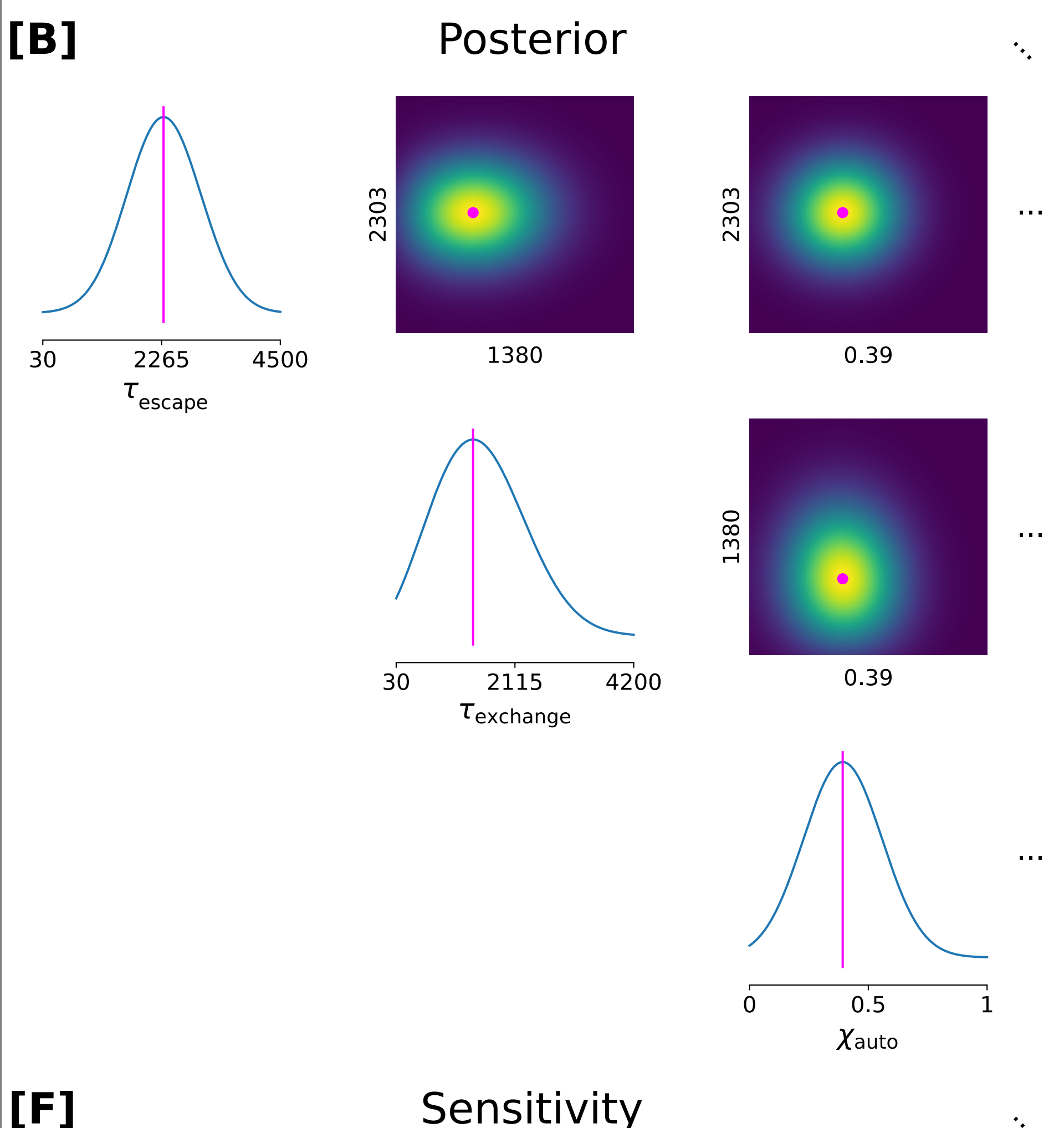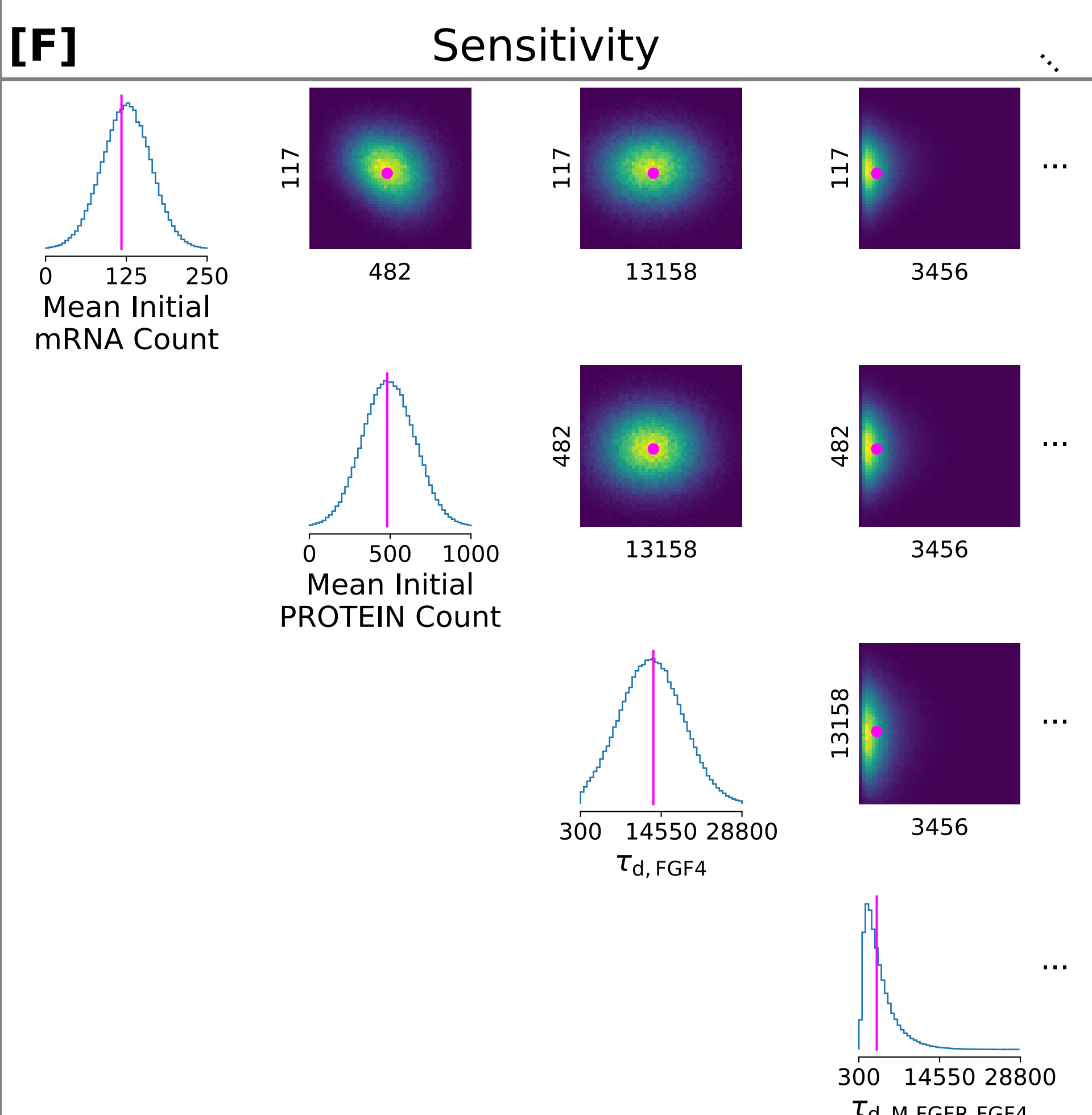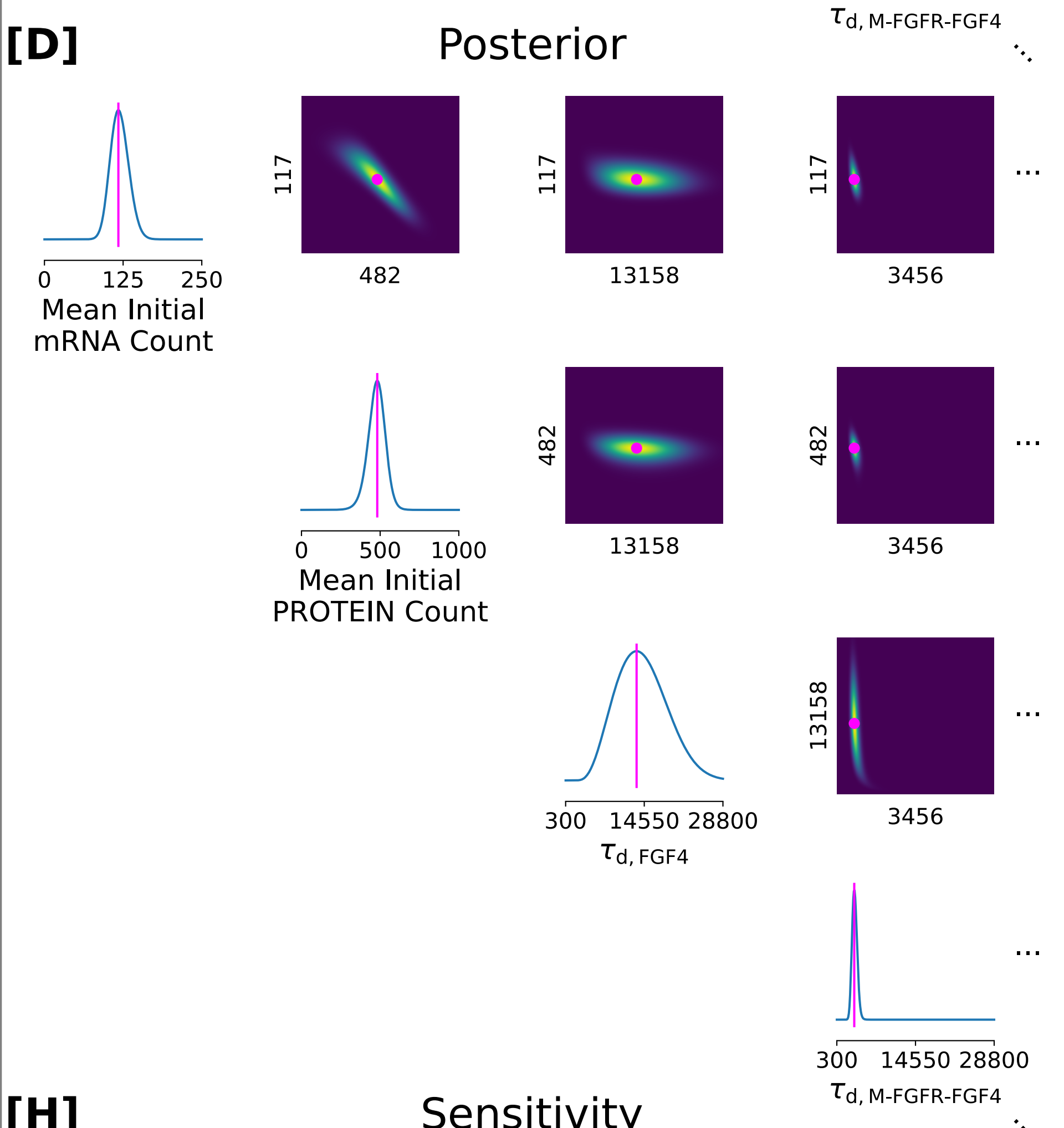

Supplement: S2 Fig — The central component of our inference scheme is the sequential neural posterior estimation (SNPE) algorithm. Both unconditional (top rows [A-D]) and conditional (bottom rows [E-H]) posterior parameter distributions were obtained following 8 consecutive rounds of inference. 800 thousand composite simulations were performed for the ITWT system. For complete details of the model parameter inference procedure, see Model parameter inference framework. [A-D] Model parameter posterior distribution. For ease of visualization, the posterior was arbitrarily partitioned into four distinctive groups. We emphasize the top-right group [B, F], which displays the most important signaling model parameter interactions. [E-H] First assessment of model parameter sensitivity. These panels show the same components as in [A-D] but the posterior is now conditioned on the maximum-a-posteriori probability (MAP) estimate of the model parameters. (PDF) [file pcbi.1012473.s003.pdf]
