# Supplementary material for: AI-powered simulation-based inference of a genuinely spatial-stochastic gene regulation model of early mouse embryogenesis
Source: PLoS Comput Biol. 2024 Nov 14;20(11):e1012473. doi: 10.1371/journal.pcbi.1012473 (PMC11614244; doi:10.1371/journal.pcbi.1012473)
Supplement: Attachment — Submitted filename: Response to reviewers.pdf [file pcbi.1012473.s004.pdf]

Response to the Reviewers of the Manuscript

"AI-powered simulation-based inference of a genuinely spatial-stochastic gene regulation model of early mouse embryogenesis"

Under Consideration for PLOS Computational Biology

Michael A. Ramirez-Sierra and Thomas R. Sokolowski

July 19, 2024

We sincerely thank the editors and reviewers for their valuable time and constructive feedback. We have carefully addressed the general comments and specific concerns raised, making the necessary edits to our manuscript as detailed below.

We present a substantially revised manuscript, in which the Introduction and Discussion sections were almost completely rewritten for incorporating the reviewer's suggestions. We also introduced a new Results subsection and additional figure panels, and expanded several of the existing Results sections, for better explaining the concrete predictions and translational value of our work. We hope that with these changes our manuscript will be considered suitable for publication in PLOS Computational Biology.

Our responses to the reviewers' comments are highlighted in blue, and descriptions of the corresponding changes in the manuscript are highlighted in red.

REVIEWER 1

SUMMARY

Ramirez-Sierra and Sokolowski present an AI-powered model of inner cell mass differentiation during preimplantation development investigating, in particular, how this stochastic system enables an incredibly robust output. They manage to reproduce many of the known properties of the ICM, including the early plasticity, the critical window of FGF segregation to allow for intercellular communication, and the fascinating robustness even when scaling the ICM, to mention a few. It's an extensive computational-heavy manuscript of a highly studied system, as the long reference list also indicates. The manuscript does not add new experimental data. Unfortunately, the importance to the field and the biological significance are unclear, and new experimentally testable predictions are lacking. Based on these concerns, I unfortunately cannot recommend publication in PLOS Computational Biology.

We thank the reviewer for appreciating the scale of our work and, in particular, the stochastic character of our approach, which enabled us to reconstitute the outstanding robustness and reproducibility of ICM patterning observed in experiments via a quantitative model. It is accurate that our work does not present new experimental data, as it is purely computational, which is why we submitted it to a computational journal. As detailed below, we believe the significance of our work arises from the fact that, in contrast to previous models, our AI-parametrized model not only reproduces some of the experimentally observed system features but successfully reproduces all of them collectively, including the aforementioned robustness to biological noise arising from a mesoscopic description of the biochemical processes generating it. Furthermore, our model makes new experimentally testable predictions, which we now emphasize more, with brief additions to the Abstract and Author Summary, but in particular in the Introduction, Discussion, and the Results subsections, as detailed in our response to CONCERN 2.

CONCERNS

1. As stated in the journal's aim: "PLOS Computational Biology publishes original research that clearly demonstrates novelty, importance to a particular field, biological significance, and conclusions that are justified by the study". I can positively argue for the novelty of this study: Applying AI technology to the ICM has, to my knowledge, never been done before. Also, the conclusion (lines 697-699) is backed up by the manuscript. However, it's unclear how this study provides "unique insights" (line 698) and "deepen our understanding of the developing early mouse embryo" (line 700). All the five suggestions listed in lines 117-124 are already known. These are not new findings. Therefore, the importance to the field and its biological significance are highly unclear.

   We thank the reviewer for positively acknowledging the computational novelty of our study. Among the five main results of our work, results 2 and 4 have not been systematically nor quantitatively addressed in any previous study, to our knowledge. In fact, CONCERN 8 of the reviewer highlights suggestion 4 as a potentially exciting new insight into ICM development, which we address specifically in our reply to CONCERN 8.

   Additionally, these five main results have not been simultaneously replicated or collectively integrated by any previous model of ICM differentiation. More importantly, our approach generates single-cell and single-molecule trajectories that faithfully mirror the temporal evolution of the studied system, unlike previous ICM differentiation modeling efforts which are limited to pseudotime representations and ad-hoc representations of intrinsic biochemical noise, oftentimes added "on top" of the modeled average dynamics via arbitrary heuristic parameters. In contrast, in our model biological noise arises from stochastic biochemical mechanisms whose dynamics are goverened by parameters with a concrete biophysical meaning. If we are misinformed on this point, we would appreciate being directed to any relevant sources we may have missed.

Our work therefore fills a gap in existing theoretical and modeling techniques by incorporating stochasticity into the problem definition based on biophysical principles, explicitly respecting empirically observed developmental time limits and realistic molecular copy numbers. We make no phenomenological assumptions and use no arbitrary time or concentration units, which is not the case for existing 2D or 3D models.
These features define the distinguishing characteristics of our modeling approach, which aims to generate detailed stochastic trajectories of system dynamics with minimal reference to experimental data, or even using qualitative observations only.
We further address the biological relevance of our work in our response to CONCERN 2.

2. This study presents a versatile computational framework, but no new experimentally testable predictions are suggested. To provide value to the community, this tool should open up one or more new directions with testable predictions that have not already been carried out. What new experiments would the authors suggest? It's challenging because the ICM is already a highly studied system. Still, as the authors note on lines 701-702, the proposed framework may be used "for exploring similar controlled stochastic processes in related biological systems." The question is, which systems do the authors have in mind? And how, more precisely, do the authors imagine the presented tool can provide new, currently unavailable insights?

   We thank the reviewer for raising this important point. We now make it more explicit and clearer which findings from our computational approach can inform new experiments. Indeed, several testable predictions made by our model are already in the manuscript, but they have not been sufficiently emphasized in their respective Results subsections in the first version of the manuscript.
   Considering the technological limitations for wet-lab experiments, we believe that future *mostly* in vitro experimental studies using, for example, optogenetic tools can validate the following model predictions:

   (A) Figure 5 demonstrates that noise at the tissue level (i.e., precision of cell-fate differentiation) scales in proportion to the square root of the wild-type-like system size (number of cells), with a magnitude lower than expected for a mutant-like system in which all cells acquire their fates autonomously. This finding is not an *a priori expectation*, as the studied wild-type-like system features many nonlinear interactions that, if not counteracted, could potentially amplify the noise. To experimentally verify this computationally inferred scaling rule, one could fix (temporarily) the maximal number of cells in an experimental system and measure differentiation precision over time. This approach should allow for the observation of how differentiation precision varies with different maximal numbers of cells, thereby testing the predicted scaling relationship.

   We have augmented the subsection "Robust cell-fate proportions are independent of cell-grid size" to better highlight this testable prediction, and provided specific details and guidelines for potential wet-lab experiments.

   (B) Figure 6 illustrates the reciprocal roles of paracrine and autocrine signaling modes in maintaining robustness of cell-fate proportioning. Specifically:
   - Panel A predicts that if none of the cells can produce FGF4, the system transitions from a fully undifferentiated (UND) population to one where epiblast (EPI) cells comprise around 90% of the population in approximately 16 hours.
   - Panel B shows that if cells can only respond to FGF4 produced by other cells (no autocrine signaling), there is a quantitative decrease in proportioning accuracy compared to the wild-type system. However, the proportioning precision remains unchanged.
   - Panel C demonstrates that if cells can only respond to FGF4 they produce themselves (no paracrine signaling), there is a loss of homeostasis. Primitive endoderm (PRE) cells take longer to achieve their target proportion, and UND cells are more prevalent than in the wild-type system, reducing the EPI population.

These findings highlight the importance of both paracrine and autocrine signaling in ensuring accurate and precise cell-fate proportioning. By experimentally manipulating the modes of FGF4 signaling, these predictions can be tested to further validate our computational model.

The subsection “Autocrine- and paracrine-signaling modes play reciprocal roles in robust cell-cell communication” has been supplemented to better harmonize these testable hypotheses for intercellular signaling modes.

(C) Figure 8 demonstrates that high variability in initial conditions (number of molecules) can potentially enhance patterning accuracy without affecting its precision. If cellular initial conditions could be systematically varied and controlled—such as by overexpressing one of the competing molecular species—Panels B, C, and D predict (quantitatively) an increase in proportioning accuracy compared to the baseline scenario. These panels also illustrate the limits of this enhancement. By experimentally varying and controlling initial conditions, such as through molecular overexpression, these predictions can be tested. This would provide a deeper understanding of how initial molecular variability influences patterning accuracy in developmental processes.

A short paragraph was added to the subsection “Increased variability in initial conditions enhances developmental accuracy while sustaining its precision” to emphasize this prediction:

To test how initial-condition variability affects developmental accuracy and precision in vitro (or even ex vivo), one could utilize photosensitive reagents to control mRNA and protein activity in living cells (i.e., optogenetics) [1, 2]. This approach would allow systematic control of cellular initial conditions, for example, via overexpression of competing molecular species.

(D) Figure 9 shows that disturbances in the target fate proportion, caused by perturbing the initial conditions in a subset of the population, depend directly on the number of affected cells within the tissue, rather than their relative positions. Additionally, these disturbances are only weakly influenced by the strength of the local (single-cell) perturbations. Our model predicts that a threshold of 25% of the population is critical; beyond this point, the system’s robustness is compromised, regardless of the perturbation strength.
This prediction aligns with our previous finding, underscoring that locally (cell-specific) highly variable initial conditions may enable the system to escape naive pluripotency more easily and reproducibly. This occurs unless the initial conditions are strongly restricted or concertedly perturbed across the tissue.
By systematically varying the initial conditions in a controlled experimental setup, these predictions can be tested, further validating the robustness of our model and providing deeper insights into the factors influencing cell-fate determination.

To highlight this prediction, we included a small paragraph at the end of subsection “Cell-fate assignment remains robust when less than 25% of cells start with perturbed initial conditions”:

In perspective, testing the impact of restricting gene expression profiles for multiple cellular subpopulations of different sizes is also achievable via optogenetics [1–3]. Our approach predicts only minor deviations for final cell-lineage distributions, in terms of their accuracy and precision, when less than 25% of the initial cellular population is perturbed accordingly; see Fig 9. Moreover, we predict that these deviations depend solely on the cumulative number of perturbed cells, rather than the specific locations of perturbation.

(E) Figure 10 shows that exogenous FGF4 affects system dynamics contingent on the timing of its addition. We agree that Panel E, which demonstrates how initial addition of FGF4 to cells with suppressed FGF4 production (“TM-FGF4”) affects the final cell fate composition, effectively recapitulates an in vitro experiment [4], as already noted in our manuscript.
However, Panels F, G, and H extend these findings by predicting how FGF4 addition influences the system based on specific timing and the amount of FGF4 added. These predictions could be tested in a refined version of the experiment reported by Raina et al. (2022), where only the amount of

exogenous FGF4 is varied and the perturbation (induction) time is fixed.
Furthermore, there are no experimental counterparts for Panel A, or for Panels B, C, and D. These panels provide additional quantitative predictions for how the cell-fate composition of the wild-type system responds to the addition of exogenous FGF4. To our knowledge, this experiment has not yet been conducted, which presents another valuable opportunity to test our predictions on FGF4-based perturbations.

We have supplemented the subsection "Cell plasticity and FGF4-sensitivity are time-window dependent", following the reviewer's general advice. We have also translated our mean added exogenous FGF4 quantities from molecular counts to concentrations, making these numbers easier to relate to and verify in lab experiments.

(F) Table 4 and Figure 15 show that the effective lifetime of intracellular FGF4 does not need to be tightly regulated, as its functional role is restricted to the extracellular feedback mechanism. Nonetheless, our results provide a full prediction range for its value. While experimentally altering protein lifetimes may be challenging, particularly in vivo, there have been successful attempts at prolonging protein lifetimes in pharmacology (protein therapeutics) [5, 6]. Moreover, it is conceivable that lifetimes could be shortened through targeted enzymatic action.
These predictions offer a basis for future experimental work aimed at manipulating FGF4 lifetimes to test the reliability of our model and its implications for the regulation of cell-fate decisions.

This idea was integrated into the summary of our testable predictions within the Discussion section.

(G) Table 4 and Figure 15 also demonstrate that the effective lifetime of the FGFR1-FGF4 (receptor-ligand) complex must be tightly regulated. We provide a full prediction range for this value, as well as an estimation of the optimal value for achieving ideal proportioning behavior. This prediction could potentially be tested using FRET (Förster Resonance Energy Transfer) experiments [7]. By employing FRET experiments to measure the lifetime of the FGFR1-FGF4 complex, the accuracy of our model predictions could be validated. This approach would further elucidate the critical role of receptor-ligand interactions in cell-fate determination.

This prediction is addressed in the Discussion section and is harmonized with general comments on the testability of the parameters inferred for this study.

Finally, we envision that our modeling and inference approaches can be applied to the following systems exhibiting high biochemical stochasticity, potentially exploiting this noise for adaptability and robustness of their behavior:
Human blastocyst formation [8–11];
Bacillus subtilis competence circuit [12–14];
Dictyostelium discoideum cell-type proportioning [15].

We have made several changes and reformatting in the Introduction and Discussion sections to correctly reflect these ideas.

3. The authors emphasize several times the spatial-stochastic capabilities of their model (it's even part of the manuscript title, and line 97 says "under truly stochastic conditions"). Still, they present a static 2D model. ICM cells (and cells in general) are highly 3D. They move around, divide, and change how many cell neighbors they have, the distance to them, and who their neighbors are. This introduces a whole new level of spatial-stochasticity that has been captured before (as far back as refs. 2 or 11). This undermines the selling point of this manuscript. Can the model presented here work in a dynamically changing 3D world to capture and build predictions for other systems than what can be carried out in a dish?

   We agree with the reviewer that there are existing models of early mouse development that incorporate spatially detailed force interactions among cells. We have acknowledged this in our Discussion section as one of the current limitations of our approach.

However, those models [16–19] lack a truly stochastic description of cell-level gene network dynamics and tissue-level molecular interactions. Consequently, they are not well-suited to study the effects of noise arising from small molecule counts and genetic expression mechanisms, which are critical in this context, and explicitly addressed in our work.

We argue throughout the Introduction, Results, and Methods sections that to properly quantify biochemical noise effects in ICM development, it is essential to bridge spatial coupling of cells with a stochastic description of intracellular dynamics. This includes accounting for mRNA and protein production noise, as well as not assuming instantaneous intercellular communication. For these reasons, we use the term *spatial-stochastic* for our modeling approach.

Unlike quoted models, our approach employs the Reaction Diffusion Master Equation (RDME) framework, describing the stochastic biochemical dynamics of signaling, gene regulatory activation and inhibition, mRNA transcription, protein translation, cytoplasmic escape, and receptor binding. These steps introduce delays in cellular responses, chiefly affecting the system behavior, as already documented in our Methods section.

This increased model fidelity comes at a high computational cost. Each *virtual* cell in our system must fire approximately 7 million reactions or events to simulate the 48-hour intra- and intercellular trajectories for all the relevant biochemical species. Not that this requires orders of magnitude more computational effort than, e. g., classical ODE integration in non-explicitly stochastic models.

Our minimal approach regarding cell-cell mechanical/force interactions can be justified beyond these high computational demands. While mechanical constraints like a growing blastocoel and TE tissue influence ICM lineages, the proportioning process remains robust in laminar, in vitro, and organoid systems, suggesting a heavy reliance on biochemical signaling [4, 10, 19, 20].

Moreover, to assess the effects of cell divisions, tissue remodeling, and extrinsic noise sources like the blastocoel and TE, a spatial-stochastic gene regulatory model must first account for settings with minimal extrinsic variability. Our model serves as a crucial stepping stone to quantify the role of noise in this system, primarily due to the stochastic nature of biochemical reactions.

Integrating detailed gene regulatory networks and cell-cell communication dynamics with richer force interactions, including cell divisions and other embryo components, is part of our roadmap. This goal is challenging and resource-intensive, making our current model a vital step towards more comprehensive approaches, and we constantly reach out for potential collaborations to advance this research.

While we acknowledge the importance of modeling dynamic 3D environments where cells move, divide, and change neighbors, our current model focuses on providing a detailed mechanistic understanding of gene regulation under stochastic conditions and non-instantaneous signaling. The static 2D model serves as an initial step to validate our approach and its predictions in a controlled setting. Future iterations of our model will incorporate dynamic changes and 3D spatial configurations to capture the full complexity of ICM cell behavior and extend our predictions to other systems beyond in vitro conditions.

To better emphasize why we use the term *spatial-stochastic* for our approach, we have modified the title to explicitly refer to *gene regulation*, clarifying this for researchers not familiar with the RDME formalism. The Introduction section has also been revised to be more transparent about our assumptions, conditions, and motivations for using a static grid of cells.

4. I tried to install and run the GitHub code provided. The scripts are pedagogically named with figure numbers. Unfortunately, I failed. Apparently, the current version of SciPy isn't working, so I downgraded to the version listed in the requirement file provided. Even then, by testing Fig6.py and Fig8.py, I get a "min() arg is an empty sequence" ValueError. I'm likely making a simple mistake. That said, please provide better guidance on how to run the presented scripts and reproduce the figures. Please provide the remaining analysis data files necessary to check the study's validity.

   We understand the inconvenience in running the figure-generating scripts and apologize for any confusion. At this stage, we have publicly disclosed our code but not yet the full dataset it operates on, due to its significant size (approximately 500 GB compressed). Consequently, the figure scripts do not work without the corresponding simulation data. However, we have granted access to parts of the dataset as desired by the reviewer. Given the size of the full dataset and its use in related work, we prefer to disclose it completely after confirmed acceptance.

   We updated our GitHub code repository to clarify the project structure and include a note explaining that the full dataset is not yet in the repository but will be provided upon reasonable request. This note will be replaced by a link to the full data repository upon acceptance. In the meantime, you will find in our GitHub repository the corresponding link to a partial dataset that can be used to regenerate most of the manuscript figures but with fewer simulation samples: `https://github.com/MARS-FIAS/Article_Modeling_Mouse_ICM.git`.

5. The model is very complicated and not particularly minimal, with 24 fixed parameters (Table 3) and 19 free parameters (Table 4). Are all these parameters really needed for what the authors want to communicate? Can Table 4 be used to make experimentally testable predictions?

   One of our core objectives was to construct a model in which biological noise is not included in an "ad hoc" manner but arises from the biochemical mechanisms at the base of the gene-regulatory network driving ICM differentiation. This approach requires explicit modeling of gene activation and repression, including transcription factor (TF) concentration-dependent promoter-state fluctuations. These alone are governed by three parameters (TF binding rate, TF unbinding rate, number of binding sites) *per regulatory interaction*. Additionally, we opted to model both mRNA transcription and protein translation, further increasing both the number of biochemical species modeled and model parameters. This was necessary because experimental numbers for mRNA abundance of some biochemical species are available, while protein numbers are not. Notably, the number of mRNA molecules produced on average is a key determinant of the biological noise arising from gene expression dynamics.
   On the other hand, our model deliberately simplifies certain interactions to minimize its complexity, such as the enzymatic action link of active FGF receptors on ERK proteins. While this process is known to involve intermediate components (RAS, RAF, etc.) within the ERK signaling cascade, we figured that replacing the entire cascade with an effective interaction would not significantly alter system behavior, given the comparably short response time of ERK to external FGF4 [21]. The fact that we can explain system behavior faithfully without explicitly incorporating the ERK cascade confirms the validity of this simplification.
   Furthermore, in contrast to existing models [16–19], we achieve richer trajectories of system dynamics with a smaller number of model parameters. Some of those other models often require more parameters and variables (close to 70, excluding force interactions), which were mostly manually tuned to incorporate some ad hoc notion of noise. While our approach includes many fixed and free parameters, significant effort was made to avoid over-engineering the model and to achieve target dynamics given several system constraints. This also pays tribute to the significantly increased computational demand of the class of models that we employ as compared to simpler models and as we already explain further above. Our detailed reasoning and arguments for our modeling choices are provided in the Methods sections.

In this respect, our model is minimal but sufficiently comprehensive to achieve our main aim of modeling how biological noise arises in the ICM from intrinsically stochastic gene-regulatory interactions and biochemical signaling processes.
As stated in our reply to CONCERN 2, Table 4, together with Figure 15, can indeed be experimentally validated, as they provide testable predictions for important parameter values that directly affect the underlying system. Importantly, these predictions include not only parameter point estimates but also interval/distribution estimates, making them suitable for experimental testing.

No additional changes were made in relation to this concern. The topic of testable predictions has been covered in our response to CONCERN 2, and our Methods section already provides an extensive description of our modeling and parameter choices.

6. Why is AI needed for what the authors try to achieve? Please elaborate.

   In our reply to CONCERN 5, we already motivate and discuss the number of parameters included in our model. As stated in the respective Results and Methods subsections, inferring such multi-parameter biophysics-inspired mechanistic models is challenging due to two main reasons: high computational demands, and scarcity of highly granular experimental data covering all involved biochemical species across relevant spatio-temporal scales. While rich experimental datasets exist for the mouse ICM, these typically involve bulk experiments that do not disentangle and track individual trajectories for all crucial system variables, simultaneously at cell- and tissue-scale. Without this granularity, a purely data-driven inference approach is essentially unfeasible.
   By employing a state-of-the-art machine learning method that implements generative techniques, we can circumvent the lack of granular data. As discussed in the Results and Discussion sections, conventional methods for parameter estimation are insufficient in this context because they are computationally highly inefficient. To infer the parameter values presented in Table 4 and the distributions shown in Figure 15, we required approximately *only* 1 million simulations to estimate the posterior distribution. Conventional methods struggle with high-dimensional models, as they cannot efficiently learn relations in parameter space without running prohibitively large numbers of simulation instances [22, 23].
   AI, specifically machine learning, provides the necessary computational efficiency and ability to manage high-dimensional parameter spaces. By leveraging generative techniques, we can efficiently explore the parameter space and derive meaningful inferences despite the limited granularity of available experimental data. In fact, our work demonstrates that the employed novel AI-powered SBI framework can achieve this even when only (essentially) one-dimensional summary statistics that mathematically encode observed experimental behavior are available. This approach allows us to achieve robust and reliable parameter estimates that would be almost impossible to obtain using traditional methods.
   In brief, these correlation analyses are computationally unfeasible with conventional approaches, as the given number of model parameters would require millions, perhaps billions, of additional simulations to properly estimate these complex synergies in high-dimensional parameter spaces [22, 23].

   To strengthen our case for using an AI-powered inference method, we have added a new Results subsection: “Posterior distributions approximated via SBI reveal correlations and potential compensatory mechanisms between model parameters”. In this addition, we exemplify how an artificial neural network surrogate allows us to uncover compensation mechanisms among parameter pairs. This result is a minor adaptation of an extensive study comparing the AI-powered inference framework against a strategy inspired by a classical optimization algorithm; which is available online and will be submitted for publication soon [24].

7. What boundary conditions are the authors applying to the 2D grid of cells?

   While there is no direct counterpart for "boundary conditions" in the RDME (Reaction-Diffusion Master Equation) simulation framework as compared to explicitly solving a system of differential equations, the flux of FGF4 escaping a single-cell region is appropriately adjusted according to the number of cellular neighbors and their specified geometry.

   In simple terms, once a signaling molecule escapes the cytoplasm, it can only reach cellular membranes–either the membrane of the origin cell or the membrane of a neighboring cell. For cells located around the edges of the tissue, this assumption mirrors a reflecting boundary condition.

   Note that while our model does not represent diffusive FGF4 leakage out of the system, it does feature several degradation channels of FGF4, namely: stochastic degradation of FGF4 in the cytoplasm and stochastic degradation of FGF4 associated with the membrane.

   This aspect is already covered in the Methods subsection "Model at Tissue Scale", where we explain how we split the outgoing FGF4 flux leaving an FGF4-producing cell and why it can be seen as similar to a reflecting boundary condition. We found it more suitable to clarify this aspect in the Methods section, rather than in the Introduction and Results sections.

8. Lines 19 and 122 (statement 4) are potentially exciting and new. Please elaborate on those.

   This is a direct continuation of our reply to CONCERN 2. Within our list of results, number 4 (number 5 in the new version) is one of the most important findings of our work, and it has been acknowledged in the Abstract and Results sections. We argue about the beneficial effects of biochemical noise with respect to the initial availability of relevant molecular resources, particularly mRNA and protein levels associated with Nanog and Gata6 genes. While the role of noise in development has been discussed in the literature [13, 25, 26], no attempts using genuine spatial-stochastic conditions have been made to address the implications of noise in the context of early mammalian embryo gene regulation, specifically ICM differentiation. As mentioned multiple times, current modeling techniques do not exploit genuinely stochastic frameworks, so the consequences of noise arising from stochastic biophysical processes such as gene expression, protein production, and (stochastic) delays between biochemical steps are not effectively captured.

   This limitation is understandable given the computational demands of simulating systems at this level of detail, especially when considering spatial aspects and diffusion. However, with the advent of generative models powered by AI, it is now possible to train surrogate simulators of biological systems that faithfully mimic high-dimensional trajectories tracking molecular copy numbers over realistic time frames at single-cell granularity.

   Exploiting these advanced techniques, we observed under our virtual-cell conditions how attaining the system's main purpose (reaching the proper cell-fate ratio) can benefit from relaxing the initial gene expression distribution and making it completely independent for each individual simulated cell. As discussed in the respective Results section, such early gene expression heterogeneity may serve as a mechanism to ensure that cells within a population are not all at the same level of potency/plasticity. This variability grants robustness to perturbations (improved adaptability) and helps achieve the desired fate proportions within the correct developmental time window. While similar effects have been observed in other biological systems [13, 25–27], here we report this, to our knowledge for the first time, for EPI-PRE proportioning in the ICM using truly spatial-stochastic conditions for gene regulation, as we now also argue in the manuscript.

   We improved the Results subsection "Increased variability in initial conditions enhances developmental accuracy while sustaining its precision", also explaining more clearly that this result is a testable prediction.

REVIEWER 2

GENERAL COMMENTS

This exhaustive treatise provides a mathematical-computational reconstruction of the 2nd lineage specification in mouse, based on the NANOG-GATA6-FGF4 signaling axis that specifies the distribution of pluripotent ICM cells into epiblast (EPI) versus primitive endoderm (PrE) lineages. The model essentially mirrors the self-organization and patterning that has been well described in the literature (eg, Zernicka-Goetz, Rossant, and Tam labs) and more recently characterized by single cell RNAseq profiling.

We thank the reviewer for appreciating the important characteristics of our model. In contrast to previous attempts, our approach is capable of simulating large ensembles of stochastic expression trajectories at the individual cell level, using faithful, "mechanistic" model implementations of the biochemical processes generating them. This can be seen as a virtual counterpart to scRNA-seq technology and, even more directly, to smFISH output trajectories.
While experimental data typically comes from bulk experiments, our model can generate such trajectories in a spatially coupled scenario within well-defined tissue geometries, where single cells communicate by exchanging signaling molecules. This is particularly pertinent to the ICM system we consider, where correct patterning critically depends on local cell-to-cell signaling via FGF4 [4]. Additionally, our simulations provide access to the full temporal dynamics of expression patterns with, essentially, arbitrarily fine resolution, resolving all individual reaction events if desired. Moreover, they can simultaneously simulate the dynamics of mRNA molecules and proteins using actual copy number counts, which are typically not accessible in standard scRNA-seq experiments.
Taken together, our simulation framework can be seen as generating in-silico data that strongly resembles scRNA-seq data, but it offers the potential to create richer datasets. It can grant access to more variables and their full dynamics when correctly parametrized and calibrated.

1. Problem formulation: FGF4 signaling through FGFR1, ERK, and FOXO (not mentioned) is a key element of this GRN, and in this manuscript provides essentially a biological framework for multiscale modeling and simulation. It would be useful to provide some background into the translational value of modeling this period in mouse (E2.5 - E4.5) and human development. The work is a seminal piece in terms of technological achievement, but from a practical perspective what is the need for mechanistic understanding of developmental liabilities during this period? For example, do adverse outcomes show up as peri-implantation failure, developmental incompetence, vulnerability to developmental toxicants, pregnancy complications? Does it offer value for improving IVF? Does it offer value in engineering 3D microsystems (blastuloids, gastruloids)? Can it advance scientific understanding of in vitro embryonic stem (ECS) or induced pluripotent stem cell (iPSC) models, particularly with regards to naive, primed, determined states of ESC differentiation, developmental programming, and canalization?

   We thank the reviewer for recognizing the potential applicability of our framework to important areas such as human fertilization and stem-cell therapies. Understanding the intricate mechanisms of early development, such as those from E2.5 to E4.5 in mouse, can indeed provide insights into various critical aspects of human development, such as the following ones:
   - Peri-implantation failure: By understanding the signaling pathways and gene regulatory networks (GRNs) involved, we can identify factors contributing to implantation success or failure.
   - Developmental incompetence: Mechanistic models help elucidate the reasons behind developmental arrest or anomalies, improving diagnostic and therapeutic strategies.
   - Vulnerability to developmental toxicants: Detailed models can predict how early developmental stages respond to environmental stressors, aiding in the assessment of teratogenic risks.
   - Pregnancy complications: Insights into early embryonic development can inform interventions to prevent or manage complications arising from developmental defects.

- Improving IVF: By refining our understanding of the factors driving successful embryo development, we can enhance assisted reproductive technologies.
- Engineering 3D microsystems (blastuloids, gastruloids): Mechanistic models can guide the creation of advanced in vitro systems that mimic early development, useful for research and therapeutic purposes.
- Advancing ECS or iPSC models: Detailed understanding of gene expression dynamics and signaling pathways can improve the use of stem cells for regenerative medicine, particularly in defining and controlling the naive, primed, and determined states of ESC differentiation.

Moreover, studying self-renewal, potency, and stem reprogramming with highly detailed models that faithfully represent biochemical noise sources offers significant benefits for treating human diseases. Our implicit interest is to uncover general developmental mechanisms and principles that are relevant across various biological systems despite noisy conditions.

We also thank the reviewer for pointing out the involvement of FOXO transcription factors in early mouse embryogenesis. Upon reviewing the literature, we found that while the FOXO family plays a crucial role in cell apoptosis and homeostasis, it is not directly involved in the second cell-fate decision driven by Nanog and Gata6, which we model in our work. Consequently, previous models of this specific network also do not account for any specific FOXO transcription factor. We appreciate the reviewer's suggestion and would be grateful if they could direct us to any relevant literature that might suggest a more direct role for FOXO in this context.

Based on the reviewer's suggestions, we have now clarified the value that theoretical modeling of mouse embryonic systems can have for enhancing the mechanistic understanding of human embryo development, particularly in terms of gene expression and signaling. The Introduction and Discussion sections have been revamped to explicitly account for the value that models such as ours can have for uncovering useful insights into human medicine, pointing out many of the aspects summarized above. We provide a holistic and concise description of the potential translational value of our modeling-inference approach for not only human biology but other relevant systems.

2. The computational framework is a 2D lattice where individual cells are represented as a 'voxel' (I thought voxel referred to volume units; please explain the computational representation a little better, and whether it is truly a voxel dimension or multi-pixels. Also, can they please comment on why a 2D lattice was used to represent a 3D ICM (computational cost?).

In our current work, the term "voxel" is indeed used to refer to individual volume units, which we assume to be constant and static. Each voxel represents a single cell. In the current version of our framework presented here, we do not model force interactions between cells or cell movement, and the biochemical simulation technique that we employ assumes well-mixed conditions at the single-cell level. Consequently, we do not specify any geometric shape for the simulated cells, as only the numerical value of the volume is relevant in our simulation scheme. Conceptually, it is fair to imagine a planar lattice of regularly spaced cells in contact, representing the effective geometry for intercellular signaling in our model.

Future iterations of our model will relax the assumption of static and immobile cells. We plan to incorporate cell force interactions, cell movements, and cell divisions, which will account for changing volumes in the biochemical scheme.

We also address the motivation for using a 2D lattice instead of a 3D representation in detail in our response to CONCERN 3 of REVIEWER 1. Briefly, the choice of a 2D lattice was primarily driven by computational cost considerations. Modeling a 3D ICM would significantly increase the computational complexity and resource requirements. The 2D representation allows us to capture the essential aspects of intercellular signaling and patterning while maintaining computational feasibility. Additionally, the 2D lattice effectively represents the nature of early developmental stages in the ICM, which can be extrapolated to 3D scenarios in future studies.

We have improved our description of the simulation geometry, which mimics a monolayer or planar 2D cellular culture and is directly relevant for intercellular signaling, as described in the Methods section. To visually aid our readers, we have also updated Fig 1 to show better what we mean by *voxel* and *2D*.

3. The SNPE algorithm provides a stochastic simulator to parameterize a desired system behavior. This is really interesting and they might relate the advantages of this approach to the evolutionary algorithm used to stochastically model shape homeostasis in a computer 'virtual embryo' model of early development (Andersen et al. (2009) https://pubmed.ncbi.nlm.nih.gov/19199386/ or other kinds of morphogenetic algorithms.

   To start with, we would like to clarify how the algorithms used in our work relate to each other: The Sequential Neural Posterior Estimation (SNPE) algorithm forms the basis of the Simulation-Based Inference (SBI) package developed and implemented by the Macke lab in Tübingen [28]. We use this package to construct our inference workflow, wrapping it around our custom (self-developed) spatial stochastic simulator of ICM gene regulation and signaling. This simulator, in turn, is developed from the Reaction Diffusion Master Equation (RDME) formalism commonly used for spatial-stochastic biochemical simulations.
   Combining these numerical frameworks accentuates their respective advantages: Our RDME-based spatial-stochastic simulator enables faithful simulation of the biochemical noise sources affecting ICM specification and the regulatory mechanisms controlling them. The SNPE-based inference workflow wrapped around the simulator allows for efficient parameterization of the spatial-stochastic model.
   We thank the reviewer for pointing out the paper by Andersen et al. (2009). This work considers many important aspects of embryo development, particularly the morphology of embryonic cells, within the context of evolutionary landscapes for lineage-guided morphological robustness, stability, and the emergence of self-repair capability. It highlights the critical importance of cell-cell communication mechanisms and the significance of cellular signaling in controlling gene expression processes for successful embryo development. Similar to our approach, the "virtual" cells of Andersen et al. occupy static positions on rectangular grids, but incorporate notions of cell division, growth, and apoptosis.
   However, the framework of Andersen et al. differs in several important aspects from ours. Their focus is on providing a platform for studying the simplest living evolutionary systems and their links with morphological homeostasis mechanisms, allowing some phenomenological and deterministic understanding of cell-cell communication and gene regulation processes as a byproduct. In contrast, our approach provides a detailed mechanistic and stochastic understanding of cell-cell signaling, gene regulatory processes, and noise sources relevant in mouse ICM specification.
   Moreover, Andersen et al. match the target outcomes (or shapes) of their virtual embryos using an evolutionary search technique, a general and popular stochastic inference method useful for phenomenological/deterministic models. However, for biophysics-inspired models like ours, these traditional techniques are often computationally inefficient because they require numerous expensive simulations to scan the model parameter space, and lack powerful parameter-space interpolation strategies. These classical techniques become prohibitively ineffective when applied to high-dimensional spatial-stochastic models. We already discussed this problem in a broader context in the Introduction and Methods sections, particularly in the first paragraphs of the "Model parameter inference framework" subsection.
   Altogether, works like the one by Andersen et al. align more with research on fitness landscapes, sequence spaces, and evolutionary games, which is beyond the scope of our present study. Recognizing its value for developmental biology modeling, as suitably pointed out by the reviewer, we cite their paper for our future perspectives in the Discussion section. Accordingly, we mention it as a potential guiding approach for extending our laminar representation into a full 3D model, in-

cluding force interactions among cells. Moreover, addressing the same context, we have prepared an extensive study comparing the AI-powered inference framework against a strategy inspired by a classical optimization algorithm; this companion study of ours is available online and will be submitted for publication soon [24].

Following this general advice from the reviewer, we have added a short sentence portraying the work by Andersen et al. as an inspiring venue for including extrinsic factor such as cell division, population growth, and apoptosis:

Several approaches addressing tissue remodeling problems have already been proposed; thus, we will focus on exploiting several of these computational platforms, such as the ones described in [29] and [30], suitable for exploring shape homeostasis emergence in simple 3D systems.

4. A general comment on verbal quality. The text is detailed and clearly written, but the large numbers of acronyms make the reading a bit cumbersome, especially the 'comparative analysis' section. I realize the practical value in defining technical terms in this already lengthy manuscript (50 pages of single line text); however, it is distracting that several abbreviations (eg, ITWT, RTM) are defined multiple times in the manuscript. Please check these and other cases of acronyms and define where needed. One example of an appropriate re-definition is in the legend for Figure 2.

   We thank the reviewer for pointing out ways to increase the clarity of our manuscript. We have carefully reviewed the text and made changes accordingly, enhancing the readability of the manuscript.

   Concretely, we have ensured that each acronym is defined only once at its first occurrence in the manuscript for each section. Subsequent usages of the acronym will refer back to the initial definition.
   We have retained redefinitions only in instances where it enhances clarity, such as in figure legends and sections where the acronym might be encountered by readers who are skimming the manuscript.
   Moreover, we have specifically reviewed the "Comparative Analysis" section to reduce the redundancy of acronym definitions and to ensure smoother readability.

5. The Methods section has several important tables on parameters at the molecular, cell and tissue levels; however, it would be helpful to add a new table to the Results (circa Line 1342) that synopsizes the dry experiment scenarios.

   Given the number of computational (dry) experiments and their need for significant context to understand their scope, we prefer to keep that information separate in subsections to avoid confusing readers with summary tables that might mislead the results. For many of these scenarios, we conduct knock-outs of components (e.g., by setting a parameter to zero). To enhance clarity without adding redundancy, we ensured that each dry experiment scenario is clearly linked to its corresponding figure and description in its Results subsection. This approach maintains the detailed context necessary for understanding each experiment while keeping the manuscript organized and focused.
   Furthermore, the purpose of Figure 2 and the existing tables in the Methods section is to provide a concise description of the two main systems discussed in our work: the biologically relevant (ITWT) and the hypothetical, entirely cell-autonomous system without spatial coupling via FGF4 (RTM). These tables are designed to give readers a clear and organized overview of the parameters at various levels (molecular, cell, and tissue).

   To provide more clarity on the differences between ITWT and RTM, we have enhanced Fig 2 with explicit parameter names and two additional panels.

6. Discussion: the opening 3 paragraphs of the Discussion are essentially rehashing introductory information (along with redefining acronyms) that could instead be part of a problem formulation statement. I would recommend that the Discussion opens with a paragraph that summarizes the key findings in the order that they will be discussed.

   We thank the reviewer for this constructive remark. We have now significantly rearranged the order of paragraphs and the flow of information in our Discussion section in order to improve clarity and coherence.

   To enhance readability and facilitate understanding for prospective readers, we have moved the problem statement and experimental-theoretical context to the beginning of the Discussion. This approach helps set the stage for the subsequent detailed analysis of our main results. Additionally, this revised structure better highlights the translational value of models like ours for gaining insights into human biology.
   The Discussion now also includes a paragraph that provides a clear summary overview of our key findings, which we furtheron discuss in more detail in the same order.

SPECIFIC COMMENTS

1. The paper is based on mouse, but the first few paragraphs of the Introduction do not explicitly state this even though they define E2.5 - E4.5. In so doing, they should also define 'Embryonic day (E)' as used in the timeline.

   We have revised the Introduction to explicitly state already in the first paragraph that our study is based on mouse embryonic development. Additionally, we now define "Embryonic day (E)" as used in the timeline. These changes ensure that the context of our study is clear from the outset.

   See the reformatted Introduction section.

2. Figure 4 and corresponding text: the simulated expression profiles in Figs 4D, 4E, and 4F are impressive. The authors simply state that this "mirrors experimental findings" and cite [19, 30, 84, 88]. Please indicate which of those findings, if any, profiled cell clusters by single cell RNA-seq (scRNA-seq), and if so can the authors add something more than merely citing these references to support their assertion of molecular recapitulation.

   We thank the reviewer for raising this point. In fact, none of those studies make use of scRNA-seq; we found only a few scRNA-seq datasets exploring gene expression for EPI-PRE specification [31, 32]. To better connect our correlation analysis to experimental findings, we are now more explicit about the experimental techniques used for characterizing these relationships, while updating our references for interested readers.

   The Results subsection "Expression profiles reveal strong linear correlations among key regulatory proteins" has been updated to better reflect the connection between the simulation results and experimental findings.

3. Lines 403-406: more context is needed to introduce the autocrine/paracrine modes before delving into the computational details.

   We have revised the manuscript to provide more context and clearly introduce the respective biological communication mode (autocrine, paracrine, and intermembrane ligand-exchange) before delving into the computational details. This ensures that readers have a better understanding of these central concepts and their relevance to our study before encountering the technical aspects without the need to read the Methods section.

The Results subsection “Autocrine- and paracrine-signaling modes play reciprocal roles in robust cell-cell communication” has been updated to reflect the reviewer’s advice by adding the following paragraph:

To dissect the roles of different communication modes in ICM specification, we modified the ITWT model to interrupt specific components of the signaling pathway. This model incorporates three distinct communication modes between neighboring cells: autocrine, paracrine, and intermembrane ligand-exchange (akin to juxtacrine) signaling via FGF4. Autocrine communication occurs when a cell secretes signaling molecules that bind to receptors on its own membrane, thereby regulating its own activity. Paracrine communication, in contrast, involves the release of signaling molecules that act on nearby cells within the local environment, facilitating coordinated responses among neighboring cells. Intermembrane ligand-exchange occurs between adjacent cells when signaling molecules unbinding from the membrane of one cell are caught by receptors on the membrane of the neighboring cell.

4. Figures 6 and 7 legends: please define the colors used in the graphs.

   We have revised the figure captions for Figures 6 and 7 to explicitly define the colors used in the graphs. This addition ensures that the figures are more accessible and comprehensible to readers.

   See corresponding figure captions.

5. Please add discussion on the mouse to human extrapolation value of the models.

   We thank the reviewer for raising this important point. We have integrated an enhanced problem statement that addresses the beneficial impact of studying animal models, both experimentally and theoretically, in advancing human medicine. This discussion highlights how insights gained from mouse models can be extrapolated to understand human biology better and improve medical treatments.

   See the introductory and in particular second-to-last paragraphs of the revamped Discussion section.

6. I am not quite clear on what the authors mean in the Methods by ’biophysically-realistic mechanistic modeling for quantitative understanding. It does not seem to reflect cellular biomechanics, but rather cellular trajectories over time. Terminology along those lines in the parlance of modern scRNAseq or spatial transcriptomic studies is ’pseudotime’ reconstruction. Please explain terminology, if appropriate.

   We appreciate the reviewer’s request for clarification on our terminology. In short, while ”mechanistic” and ”mechanical” share similar notions, ”mechanistic” is a popular term in the biophysics and systems biology communities for referring to comprehensive descriptions of biological problems using physical first principles, whereas ”mechanical” refers to force interactions.
   Our use of ”mechanistic” refers to the explicit inclusion of individual biochemical steps involved in gene-regulatory processes. A ”mechanistic” model of gene regulation explicitly simulates stochastic transitions between different binding states of the promoters that drive the expression of relevant mRNA molecules. Additionally, it models the individual (single molecule) expression of these molecules, as well as individual translation events that produce single molecules of proteins. Accounting for these processes explicitly introduces biological noise in our model in a way that depends solely on biochemical parameters corresponding to real biochemical quantities. This approach avoids the need for introducing ”ad hoc” parameters for tuning the noise level, commonly used in more simplistic gene-regulatory models, including previous models of ICM specification known to us.
   In contrast, ”mechanical” refers to model features that explicitly account for cellular biomechanics and intercellular forces, which we plan to include in future iterations of our model. We now clearly

discriminate between these two terms starting from our Introduction.
However, we believe that it is not suitable in our study to use terminology such as "pseudotime reconstruction" as used in modeling/analysis approaches fitting scRNA-seq data. Our approach does not allude directly to transition steps in pluripotency or cell-differentiation landscapes. Instead, it tracks single-molecule mRNA and protein copy numbers for each single (virtual) cell over realistic time periods, using realistic values for molecular lifetimes and experimentally validated developmental windows. These trajectories ultimately produce different cell fates, but we are only modeling one such event. In that respect, the concept of "pseudotime reconstruction" would not be well suited for describing the process that we study.

The Introduction section has been revised to clarify the scope of our approach. We are now more explicit about the terminology for readers not familiar with the RDME formalism by better stating our focus: a spatial-stochastic model of gene regulation. Moreover, we are more explicit about the lack of "mechanical" or "force" interactions among cells and the motivation behind this modeling choice.

References

[1] Gautier A, Gauron C, Volovitch M, Bensimon D, Jullien L, Vriz S. How to control proteins with light in living systems. Nature Chemical Biology. 2014;10(7):533–541. doi:10.1038/nchembio.1534.

[2] Frei T, Chang CH, Filo M, Arampatzis A, Khammash M. A genetic mammalian proportional–integral feedback control circuit for robust and precise gene regulation. Proceedings of the National Academy of Sciences. 2022;119(24):e2122132119. doi:10.1073/pnas.2122132119.

[3] Perkins ML, Benzinger D, Arcak M, Khammash M. Cell-in-the-loop pattern formation with optogenetically emulated cell-to-cell signaling. Nature Communications. 2020;11(1):1355. doi:10.1038/s41467-020-15166-3.

[4] Raina D, Bahadori A, Stanoev A, Protzek M, Koseska A, Schröter C. Cell-cell communication through FGF4 generates and maintains robust proportions of differentiated cell types in embryonic stem cells. Development. 2021;148(21):dev199926. doi:10.1242/dev.199926.

[5] Urban DJ, Roth BL. DREADDs (Designer Receptors Exclusively Activated by Designer Drugs): Chemogenetic Tools with Therapeutic Utility. Annual Review of Pharmacology and Toxicology. 2015;55(Volume 55, 2015):399–417. doi:10.1146/annurev-pharmtox-010814-124803.

[6] Nies VJM, Sancar G, Liu W, van Zutphen T, Struik D, Yu RT, et al. Fibroblast Growth Factor Signaling in Metabolic Regulation. Frontiers in Endocrinology. 2016;6.

[7] Karl K, Del Piccolo N, Light T, Roy T, Dudeja P, Ursachi VC, et al. Ligand bias underlies differential signaling of multiple FGFs via FGFR1. eLife. 2024;12:RP88144. doi:10.7554/eLife.88144.

[8] Cockburn K, Rossant J. Making the blastocyst: lessons from the mouse. The Journal of Clinical Investigation. 2010;120(4):995–1003. doi:10.1172/JCI41229.

[9] Kim J, Koo BK, Knoblich JA. Human organoids: model systems for human biology and medicine. Nature Reviews Molecular Cell Biology. 2020;21(10):571–584. doi:10.1038/s41580-020-0259-3.

[10] Płusa B, Piliszek A. Common principles of early mammalian embryo self-organisation. Development. 2020;147(dev183079). doi:10.1242/dev.183079.

[11] Yeh CY, Huang WH, Chen HC, Meir YJJ. Capturing Pluripotency and Beyond. Cells. 2021;10(12):3558. doi:10.3390/cells10123558.

[12] Maamar H, Raj A, Dubnau D. Noise in Gene Expression Determines Cell Fate in Bacillus subtilis. Science. 2007;317(5837):526–529. doi:10.1126/science.1140818.

[13] Mugler A, Kittisopikul M, Hayden L, Liu J, Wiggins CH, Süel GM, et al. Noise Expands the Response Range of the Bacillus subtilis Competence Circuit. PLOS Computational Biology. 2016;12(3):e1004793. doi:10.1371/journal.pcbi.1004793.

[14] Wang Q, Zhang X, Yang Y. Effects of noise and harmonic excitation on the growth of Bacillus subtilis biofilm. Biosystems. 2021;201:104329. doi:10.1016/j.biosystems.2020.104329.

[15] Gruenheit N, Parkinson K, Brimson CA, Kuwana S, Johnson EJ, Nagayama K, et al. Cell Cycle Heterogeneity Can Generate Robust Cell Type Proportioning. Developmental Cell. 2018;47(4):494–508.e4. doi:10.1016/j.devcel.2018.09.023.

[16] Krupinski P, Chickarmane V, Peterson C. Simulating the Mammalian Blastocyst - Molecular and Mechanical Interactions Pattern the Embryo. PLOS Computational Biology. 2011;7(5):e1001128. doi:10.1371/journal.pcbi.1001128.

[17] Nissen SB, Perera M, Gonzalez JM, Morgani SM, Jensen MH, Sneppen K, et al. Four simple rules that are sufficient to generate the mammalian blastocyst. PLOS Biology. 2017;15(7):e2000737. doi:10.1371/journal.pbio.2000737.

[18] Tosenberger A, Gonze D, Bessonnard S, Cohen-Tannoudji M, Chazaud C, Dupont G. A multi-scale model of early cell lineage specification including cell division. npj Systems Biology and Applications. 2017;3(1):1–11. doi:10.1038/s41540-017-0017-0.

[19] Saiz N, Mora-Bitria L, Rahman S, George H, Herder JP, Garcia-Ojalvo J, et al. Growth-factor-mediated coupling between lineage size and cell fate choice underlies robustness of mammalian development. eLife. 2020;9. doi:10.7554/eLife.56079.

[20] Fischer SC, Schardt S, Lilao-Garzón J, Muñoz-Descalzo S. The salt-and-pepper pattern in mouse blastocysts is compatible with signaling beyond the nearest neighbors. iScience. 2023;26(11). doi:10.1016/j.isci.2023.108106.

[21] Raina D, Fabris F, Morelli LG, Schröter C. Intermittent ERK oscillations downstream of FGF in mouse embryonic stem cells. Development. 2022;149(4):dev199710. doi:10.1242/dev.199710.

[22] Gonçalves PJ, Lueckmann JM, Deistler M, Nonnenmacher M, Öcal K, Bassetto G, et al. Training deep neural density estimators to identify mechanistic models of neural dynamics. eLife. 2020;9:e56261. doi:10.7554/eLife.56261.

[23] Tolley N, Rodrigues PLC, Gramfort A, Jones SR. Methods and considerations for estimating parameters in biophysically detailed neural models with simulation based inference. PLOS Computational Biology. 2024;20(2):e1011108. doi:10.1371/journal.pcbi.1011108.

[24] Ramirez-Sierra MA, Sokolowski TR. Comparing AI versus Optimization Workflows for Simulation-Based Inference of Spatial-Stochastic Systems; 2024. Available from: `http://arxiv.org/abs/2407.10938`.

[25] Eldar A, Elowitz MB. Functional roles for noise in genetic circuits. Nature. 2010;467(7312):167–173. doi:10.1038/nature09326.

[26] Simon CS, Hadjantonakis AK, Schröter C. Making lineage decisions with biological noise: Lessons from the early mouse embryo. WIREs Developmental Biology. 2018;7(4):e319. doi:https://doi.org/10.1002/wdev.319.

[27] Holmes WR, Mochel NSRd, Wang Q, Du H, Peng T, Chiang M, et al. Gene Expression Noise Enhances Robust Organization of the Early Mammalian Blastocyst. PLOS Computational Biology. 2017;13(1):e1005320. doi:10.1371/journal.pcbi.1005320.

[28] Tejero-Cantero A, Boelts J, Deistler M, Lueckmann JM, Durkan C, Gonçalves PJ, et al. sbi: A toolkit for simulation-based inference. Journal of Open Source Software. 2020;5(52):2505. doi:10.21105/joss.02505.

[29] Andersen T, Newman R, Otter T. Shape Homeostasis in Virtual Embryos. Artificial Life. 2009;15(2):161–183. doi:10.1162/artl.2009.15.2.15201.

[30] Liebisch T, Drusko A, Mathew B, Stelzer EHK, Fischer SC, Matthäus F. Cell fate clusters in ICM organoids arise from cell fate heredity and division: a modelling approach. Scientific Reports. 2020;10(1):22405. doi:10.1038/s41598-020-80141-3.

[31] Mohammed H, Hernando-Herraez I, Savino A, Scialdone A, Macaulay I, Mulas C, et al. Single-Cell Landscape of Transcriptional Heterogeneity and Cell Fate Decisions during Mouse Early Gastrulation. Cell Reports. 2017;20(5):1215–1228. doi:10.1016/j.celrep.2017.07.009.

[32] Garg V, Yang Y, Nowotschin S, Setty M, Kuo YY, Sharma R, et al.. Single-cell analysis of bidirectional reprogramming between early embryonic states reveals mechanisms of differential lineage plasticities; 2023. Available from: `https://www.biorxiv.org/content/10.1101/2023.03.28.534648v1`.
